# Meta-Analysis of Heifer Traits Identified Reproductive Pathways in *Bos indicus* Cattle

**DOI:** 10.3390/genes12050768

**Published:** 2021-05-18

**Authors:** Muhammad S. Tahir, Laercio R. Porto-Neto, Cedric Gondro, Olasege B. Shittu, Kimberley Wockner, Andre W. L. Tan, Hugo R. Smith, Gabriela C. Gouveia, Jagish Kour, Marina R. S. Fortes

**Affiliations:** 1 School of Chemistry and Molecular Bioscience, The University of Queensland Australia, Brisbane, QLD 4072, Australia; m.tahir@uqconnect.edu.au (M.S.T.); b.olasege@uqconnect.edu.au (O.B.S.); k.wockner@uq.edu.au (K.W.); weiliangandre.tan@uqconnect.edu.au (A.W.L.T.); h.rosssmith@uqconnect.edu.au (H.R.S.); jagishkour@uq.net.au (J.K.); 2 Commonwealth Scientific and Industrial Research Organization, Brisbane, QLD 4072, Australia; laercio.portoneto@csiro.au; 3 Department of Animal Science, Michigan State University, East Lansing, MI 48824, USA; gondroce@msu.edu; 4 Animal Science Department, Veterinary School, Federal University of Minas Gerais, Belo Horizonte 31270-901, Brazil; gabrielacgouveia@hotmail.com

**Keywords:** GWAS, meta-analysis, gene ontology, *Bos indicus*, Brahman cattle, fertility, puberty, hypothalamus, pituitary, ovary, biological pathways

## Abstract

Fertility traits measured early in life define the reproductive potential of heifers. Knowledge of genetics and biology can help devise genomic selection methods to improve heifer fertility. In this study, we used ~2400 Brahman cattle to perform GWAS and multi-trait meta-analysis to determine genomic regions associated with heifer fertility. Heifer traits measured were pregnancy at first mating opportunity (PREG1, a binary trait), first conception score (FCS, score 1 to 3) and rebreeding score (REB, score 1 to 3.5). The heritability estimates were 0.17 (0.03) for PREG1, 0.11 (0.05) for FCS and 0.28 (0.05) for REB. The three traits were highly genetically correlated (0.75–0.83) as expected. Meta-analysis was performed using SNP effects estimated for each of the three traits, adjusted for standard error. We identified 1359 significant SNPs (*p*-value < 9.9 × 10^−6^ at FDR < 0.0001) in the multi-trait meta-analysis. Genomic regions of 0.5 Mb around each significant SNP from the meta-analysis were annotated to create a list of 2560 positional candidate genes. The most significant SNP was in the vicinity of a genomic region on chromosome 8, encompassing the genes *SLC44A1*, *FSD1L*, *FKTN*, *TAL2* and *TMEM38B*. The genomic region in humans that contains homologs of these genes is associated with age at puberty in girls. Top significant SNPs pointed to additional fertility-related genes, again within a 0.5 Mb region, including *ESR2*, *ITPR1*, *GNG2, RGS9BP, ANKRD27*, *TDRD12*, *GRM1, MTHFD1, PTGDR* and *NTNG1.* Functional pathway enrichment analysis resulted in many positional candidate genes relating to known fertility pathways, including GnRH signaling, estrogen signaling, progesterone mediated oocyte maturation, cAMP signaling, calcium signaling, glutamatergic signaling, focal adhesion, PI3K-AKT signaling and ovarian steroidogenesis pathway. The comparison of results from this study with previous transcriptomics and proteomics studies on puberty of the same cattle breed (Brahman) but in a different population identified 392 genes in common from which some genes—*BRAF, GABRA2, GABR1B, GAD1, FSHR, CNGA3, PDE10A, SNAP25, ESR2, GRIA2, ORAI1, EGFR, CHRNA5, VDAC2, ACVR2B, ORAI3, CYP11A1, GRIN2A, ATP2B3, CAMK2A, PLA2G, CAMK2D and MAPK3*—are also part of the above-mentioned pathways. The biological functions of the positional candidate genes and their annotation to known pathways allowed integrating the results into a bigger picture of molecular mechanisms related to puberty in the hypothalamus–pituitary–ovarian axis. A reasonable number of genes, common between previous puberty studies and this study on early reproductive traits, corroborates the proposed molecular mechanisms. This study identified the polymorphism associated with early reproductive traits, and candidate genes that provided a visualization of the proposed mechanisms, coordinating the hypothalamic, pituitary, and ovarian functions for reproductive performance in Brahman cattle.

## 1. Introduction

Early reproductive traits contribute to the fertility of female beef cattle. These traits affect the cows’ lifetime reproductive capacity, which has a flow-on repercussion to farm economics [1,2]. Puberty, an early reproductive trait, is delayed in Brahman cattle (a composite cattle breed largely descended from *Bos indicus* cattle) as compared to other beef breeds, and as a consequence the first calving event is also delayed [1,3]. Timing of puberty, establishment of pregnancy and resumption of post-calving estrous cycles are important benchmarks of productivity for beef cows [1]. This criterion of beef productivity is dependent on early reproductive traits, which rely on the biological phenomenon of puberty.

Attainment of puberty is a complex coordination between ovaries, hypothalamus and pituitary through negative and positive feedback mechanisms. Ovaries through estrogen signaling maintain negative feedback on the hypothalamus to prevent secretion of GnRH before puberty [4,5]. At puberty, the negative feedback of estrogen on hypothalamus is reverted to positive feedback, which allows GnRH production. Kisspeptin, GABAergic, glutamatergic and cholinergic neurons coordinate with GnRH neurons [6,7,8,9]. These neurons, through their neurotransmitters, activate signaling cascades including calcium signaling, cAMP signaling and MAPK signaling in GnRH neurons to secrete GnRH [7,10,11,12]. Signaling by GnRH through GnRH receptor (GNRHR) causes the pituitary to release FSH and LH by activating Calcium and cAMP pathways [13,14]. In ovaries, FSH facilitates follicular growth, while LH is involved in maturation of oocyte and ovulation.

We included three early reproductive traits in our analyses: pregnancy at first mating opportunity (PREG1, a binary trait), first conception score (FCS, score 1–3) and rebreeding score based on the outcome of the first two mating opportunities (REB, score 1–3.5). These traits are influenced by the biological phenomenon of puberty and by the length of postpartum anestrous. Studying these traits can reveal the biological basis of early puberty in beef cows. Early reproductive traits are usually heritable to a low-to-moderate extent (~0.1 to 0.5), which indicates the influence of genetics and environmental factors such as location, breed and nutrition on these traits [15]. A study of PREG1 in Nellore cattle, estimated a heritability of 0.18 and reported 101 associated SNPs with a *p*-value < 0.001 and a false discovery rate value of 0.53 [16]. Post-partum anestrous interval (PPAI) and post-partum ovulation before weaning (PW), which are traits based on observing the resumption of the cycle after the first calving (biologically similar to REB in this study), have reported heritabilities of 0.26 and 0.08 in Brahman cattle, respectively [17]. A trait termed “early pregnancy” in Nellore cattle, which is similar to FCS in this study, has an estimated heritability of 0.30 [18]. These studies indicate that early reproductive traits do have a heritable component that can be explored in cattle breeding programs. However, previous GWAS revealed their complex nature: many associated polymorphisms with small effects. Small effects might be ignored in GWAS when the threshold for SNP significance is set at very low *p*-values because of multiple tests [19,20,21]. This issue results in missing heritability which is often observed for complex traits, especially when the heritability is not high [22]. Meta-analysis of complex traits can address the problem of small SNP effects [19,20,21], as it considers SNP effects across traits or populations, looking for concordant results [23]. Meta-analyses have identified higher numbers of significant SNPs for reproductive traits, as compared to single-trait GWAS [24,25]. In this context, using three traits in a meta-analysis might be more powerful than looking at PREG1, FCS and REB separately.

## 2. Materials and Methods 

### 2.1. Phenotypes

The data for this study were obtained from the “Female Fertility PhenoBank” (L.GEN.1710) project funded by Meat and Livestock Australia (MLA). This project maintains a database of reproductive phenotypes recorded in female cattle, with a focus on tropical beef breeds raised in northern Australia. Data from previous genomics projects and new industry partners were curated and gathered in PhenoBank, which is an ongoing effort to create a robust platform for cattle genomics research. We selected, curated and combined data from 2400 Brahman cows, for which we defined new phenotypes. These cow records were sourced from the Cooperative Research Center for Beef Genetics Technologies (or Beef CRC), the Northern Territory DITT breeding herd and the Kamilaroi herd investigated in a CSIRO-led project. The datasets contained enough information for us to define phenotypes based on the reproductive performance records of the first two mating seasons of each cow’s life. These phenotypes are easy to measure traits that can, in the future, be adopted by the beef industry; they do not require intensive measurement. The three early-in-life reproductive traits obtained from PhenoBank were: PREG1, FCS, and REB as per Table 1. The number of animals for each trait category, and in each dataset (see Table S1), were the numbers available after data curation based on the availability of accurate pregnancy records for contemporary groups of cows.

### 2.2. Genotypes and Imputation

Medium-density genotypes of selected cows were available from PhenoBank. The genotypes of the Beef CRC cows were already available and incorporated into PhenoBank. These cows were genotyped with a medium density SNP chip, Bovine50K v.1 [17]. Genotyping of NT DITT cows and Kamilaroi cows was recently completed by NEOGEN Australia with funding from the PhenoBank. NT DITT cows were genotyped with the medium-density GGP Bovine50K SNP chip for beef, while Kamilaroi cows were genotyped with the GGP TropBeef 35K SNP chip. Medium-density genotypes of these datasets were imputed to high-density, separately, as described below.

A reference panel of 546 Brahman animals genotyped with the BovineHD (770K) SNP chip was used to impute genotypes from the medium-density SNP panels. A combination of Eagle v2.4.1 [26] and Minimac3 [27] was used for imputation. Briefly, both, high and medium-density genotypes were mapped with the same genetic marker map (ARS-UCD 1.2 HD 2018). The genotypes of the high and medium-density datasets were first passed through quality control and SNPs with a call rate > 0.85 and MAF > 0.05 were retained for analyses. Then, all genotype sets were split into individual chromosomes, one chromosome per file, using PLINK [28]. Individual chromosome files of high and medium datasets were phased, separately, using Eagle v2.4.1. Then, Minimac3 was used to impute individually phased chromosomes of medium-density datasets to high-density using individually phased chromosomes of the reference panel. Individually imputed chromosomes of each dataset were converted to “*ped. map.”* format using vcf-tools [29], and then merged using PLINK. For quality control, imputed SNPs with allelic correlation (R^2^) value less than 0.4 were discarded [30]. As a result, 718,464 SNPs were retained for the beef CRC dataset, 719,140 SNPs remained for Kamilaroi, and 689,037 SNPs remained for the NT DITT dataset. These datasets were then combined for analyses of each trait. The combined genotypes datasets for each trait were passed through a final quality control (SNPs with a call rate < 0.9 and MAF < 0.05 were discarded) to get over 500,000 SNPs for the combined dataset. As the number of animals differed for each trait, so did the SNPs that passed the quality control (587,900 SNPs for PREG1, 584,510 SNPs for FCS, and 584,344 SNPs for REB).

### 2.3. Fixed Effects

Each phenotype dataset had its own fixed effects to account for contemporary group effects, as these were separate herds raised independently. Phenotypes of individual datasets were adjusted for their own significant fixed effects, separately, using *SNP & Variation Suite v8.x Golden Helix* [31]. Contemporary groups were defined by farm location (animals raised together on the same farm), birth year, which informs the cow crop (year) and birth month class (Aug to Nov = Class A; Dec to April = Class B). For the Beef CRC dataset, farm, cow crop and birth month class were used as fixed effects, separately. For the NT DITT dataset, cow crop and birth month class were used as fixed effects, separately. For the Kamilaroi dataset, cow crop was used as a fixed effect. The distribution of animals in contemporary groups is provided in Table S2. A previously reported method to adjust for fixed effects was used as follows [32,33]:

y = X_1_β_1_ + X_2_β_2_ + … X_n_β_n_ + Zμ + e

where X_1_.._n_ are vectors of 1..n fixed effects, β_1_.._n_ are estimates of 1..n fixed effects. Z is an incidence matrix of random polygenic effects; μ is the estimate of random polygenic effects ∼N(0, Gσμ^2^) where G is the genomic relationship matrix (GRM) based on all SNP variants. Residual is represented as “e” ∼N(0, σe^2^).

Estimates of fixed effects were obtained from the equation above and were subtracted from the original phenotype y to get the adjusted phenotype (y’). The adjusted phenotype still contained the additive random genetic effects and residual.

y’ = y − X_1_β_1_ + X_2_β_2_ + … X_n_β_n_

After adjusting for fixed effects, the three datasets were combined to make a single dataset for each trait with the adjusted phenotypes. The adjusted phenotypes (y’) for each trait were then used in all subsequent analyses.

### 2.4. Genome-Wide Association Studies

Genome wide association studies (GWAS) were done for the combined dataset of each trait using *SNP & Variation Suite v8.x Golden Helix* [31]. Adjusted phenotypes from the above equation were used in a single locus (SNP-by-SNP) GWAS. Variance components were estimated through REML for each trait, separately, in which the GRM of all animals from the combined datasets for each trait was used to estimate the heritabilities of the traits using *SNP & Variation Suite v8.x Golden Helix.* As the data in the combined datasets originated from different original datasets, the original datasets themselves were considered a fixed effect in this analysis. The following linear mixed model was used to perform GWAS for each trait:

y’ = Dβ + Zμ + Sα + e

where D is an incidence matrix of fixed effects (datasets), β is the estimate of fixed effects. Z is as previously described. S is an incidence matrix of genotypes (coded as 0, 1, or 2 copies of the minor allele) and α is the estimate of SNP effects.

### 2.5. Genetic and Phenotypic Correlation of the Traits

Adjusted phenotypes were used to estimate pairwise genetic correlations amongst the three traits using *SNP & Variation Suite v8.x Golden Helix* [31]. The analysis estimated variance components of the traits using the genomic relationship matrix through REML. The following equation was used to compute genetic correlations in trait pairs:

$$rG=\frac{C\left(G12\right)}{\;\sqrt{V\left(G1\right)\;V\left(G2\right)}}$$

where *rG* is the genetic correlation between two traits, *C*(*G*12) is the genetic covariance of the two traits, *V*(*G*1) and *V*(*G*2) are the genetic variances of the two traits.

The phenotypic correlation between the traits was calculated using the following equation [34]:

$$rP=\frac{\sigma \mathrm{uxy}+\;\sigma \mathrm{exy}}{\sqrt{(\sigma^{2}\mathrm{ux}+{\;\sigma }^{2}\mathrm{ex})\times (\sigma^{2}\mathrm{uy}+{\;\sigma }^{2}\mathrm{ey})}}$$

where *rP* is phenotypic correlation between two traits. σu_xy_ and σe_xy_ are genetic and residual covariances between two traits x and y, respectively. σ^2^u_x and_ σ^2^u_y_ are genetic variances and σ^2^e_x_ and σ^2^e_y_ are the residual variances of traits x and y.

### 2.6. Multi-Trait Meta-Analysis

Multi-trait meta-analysis was performed across three traits, using *SNP & Variation Suite v8.x Golden Helix* [31]. Meta-analysis used SNP effects (β) and their standard error (SE) from the three traits to construct χ^2^ distribution of the traits with following equation [35]:

Multi-trait *x*^2^ = *t’_i_ V*^−1^* t_i_*

where *t_i_* is a 3 × 1 vector of the *i*th SNP effects divided by their respective standard errors, *t′_I_* is the transpose vector of *t_i_*, and *V*^−1^ is an inverse of the 3 × 3 correlation matrix of the correlations between the *t*-values. Expected false discovery rate (FDR) for SNPs associations was computed as *f = αm/s*, where *m* is the total number of tests, *α* is threshold of significance, and *s* is the number of significant tests with a *p*-value *< α*. To determine the value of α, resulting in FDR lower than 5%, the procedure described by Benjamini [36] was applied. Here, *s* was defined as the rank position “*i*” with the largest *p*-value satisfying *Pi ≤ fi/m* for *f* = 0.05 [37].

### 2.7. SNP Informed Positional Candidate Gene List

Keeping in mind the extent of linkage disequilibrium (LD) in cattle [38], a list of genes within a 0.5 Mb window of significant SNPs (*p*-value < 9.0 × 10^−6^, FDR < 0.0001) was prepared. For this purpose, we used SNP and gene positions from the new reference genome reference genome (ARS_UCD1.2, GenBank assembly accession GCA_002263795.2) and ENSEMBL resources [39]. This gene list was termed “positional candidate gene list”.

### 2.8. Functional Pathway Analysis

We used the positional candidate gene list from GWAS as the target gene list to perform gene ontology and pathway analysis using DAVID [40]. DAVID first classifies the genes present in the target gene list into their respective ontologies and biological pathways. Over-representation of a set of genes from the target gene list in specific pathways is then determined as a pathway enrichment score (*p*-value), in comparison with a background gene list [40,41]. We used two background gene lists for this purpose: (1) default background list of *Bos taurus* in DAVID, which contains all annotated genes in the cattle genome; and (2) a trained background gene list. The trained background gene list consisted of genes annotated to known fertility pathways (taken from the KEGG PATHWAY database) and genes searched using “Guildify” software [42] with keywords related to fertility and puberty. The keywords were puberty, GNRH, luteinizing, follicle, ovary, hypothalamus, pituitary, calcium, estrogen, oocyte, meiosis, and progesterone.

### 2.9. Using Information from Transcriptomics and Proteomics Studies Related to Cattle Puberty

Lists of differentially expressed genes and proteins that emerged from the comparison of transcriptomics and proteomics data of hypothalamus, pituitary and ovaries of pre- and post-pubertal Brahman heifers were available from previous work of our group [43,44,45]. In these previous studies, the comparative transcriptomics and proteomics of the hypothalamus, pituitary and ovarian tissues of pre- vs. post-pubertal cattle was done by RNA sequencing and tandem mass spectrometry (MS/MS), respectively. Both transcriptomics and proteomics approaches identified candidate genes involved in the onset of puberty in Brahman cattle. The genomics approaches in current study also aimed to address early reproductive traits, involving the phenomenon of puberty. We combined the significant differentially expressed genes lists from these previous transcriptomics and proteomics experiments and named the new gene list as “transcriptomics-proteomics gene list”. This gene list was compared with the positional candidate gene list of the current study to identify common genes between similar but two independent experimental approaches. The resultant common gene list was named “common multi-omics gene list”.

### 2.10. Transcription Factor Analysis

The fertility-related trained background gene list, which we prepared for the gene ontology and pathway analysis above, was also used to identify top transcription factors for the genes involved in fertility-related pathways using PASTAA [46]. Briefly, the genes of fertility related trained background gene list were searched in ENSEMBL for human homologues. The human homologues were then used as input to the PASTAA software and affinity scores of transcription factors were determined for the input list of genes. The transcription factors with the highest affinity scores were termed as top transcription factors. The top transcription factors were cross-checked with the positional candidate gene list of this study to verify if they were also in proximity with significant SNP from this study.

## 3. Results

The accuracy of imputation, in terms of allelic R^2^, for CRC, Kamilaroi and NT DITT cows’ datasets was 0.95, 0.93 and 0.92, respectively. Linear mixed model analysis of three traits resulted in low-to-moderate heritabilities, 0.11 for FCS, 0.17 for PREG1, and 0.28 for REB. Genetic and phenotypic correlations between the three traits were positive and high (Table 2).

**Figure 1:**
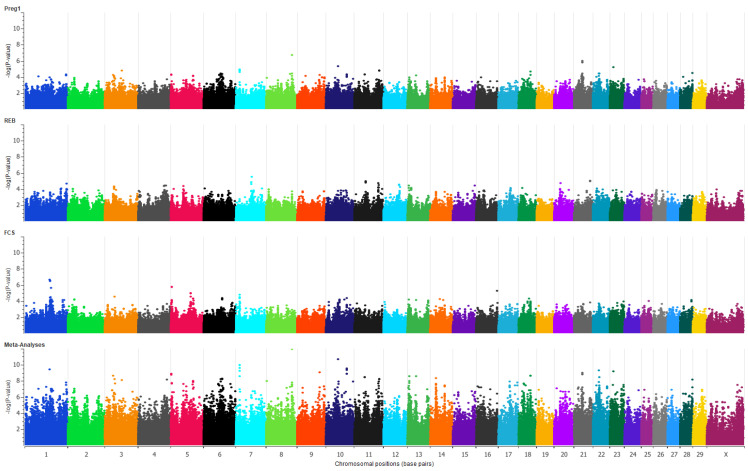
Genome-wide association studies of three early reproductive traits and their meta-analysis. Polymorphism association plots for pregnancy after first mating opportunity (PREG1), rebreeding score (REB), first conception score (FCS), and multi-trait meta-analysis are represented from top to bottom, respectively. In each plot, the *x*-axis has the chromosomal positions and the *y*-axis is the −log(*p*-value) of each SNP association.

The individual GWA studies showed a few moderately significant SNPs for each trait. In total, 59 suggestively associated SNPs (*p*-value < 9.9 × 10^−5^) were present across different chromosomes for PREG1 (Figure 1). For PREG1, the SNP with highest significance (*p*-value 2.0 × 10^−7^) was on chromosome 8. A cluster of SNPs associated with PREG1 was present on chromosome 21 with the peak SNP having a *p*-value of 1.1 × 10^−6^. GWAS results for FCS comprised of 51 SNPs above the threshold (*p*-value < 9.9 × 10^−5^) for suggestive association (Figure 1). The SNP with the highest significance for FCS (*p*-value 2.4 × 10^−7^) was on chromosome 1. Two more SNPs in the same chromosome 1 region were observed, with *p*-values 2.9 × 10^−7^ and 3.3 × 10^−7^. GWAS for REB resulted in 42 suggestive SNPs (*p*-value < 9.9 × 10^−5^) across different chromosomes, and the top five SNPs were on chromosome 7, 11 and 21 (Figure 1).

Multi-trait meta-analysis of the three traits resulted in 1359 significant SNPs with a *p*-value < 9.9 × 10^−6^ at FDR < 0.0001 (Figure 1) (Table S2). The top five chromosomes had 36.57% of all significant SNPs; they were chromosomes 1 (114 SNPs), 22 (111 SNPs), 6 (106 SNPs), 11 (87 SNPs) and 5 (79 SNPs). These SNPs association results corroborate the polygenic nature of reproductive traits.

We mined the 0.5 Mb around all significant SNPs (*p*-value < 9.9 × 10^−6^, FDR < 0.0001) and listed 2560 positional candidate genes. The most significant SNP out of the multi-trait meta-analyses was ARS-BFGL-NGS-111964 (*p*-value 1.15 × 10^−12^) mapped to chromosome 8. The genomic region of 0.5 Mb around this top SNP has six genes, *SLC44A1, FSD1L, FKTN, TAL2, TMEM38B* and *U6*. Chromosomes 3, 5, 7, 9, 10, 21, 22, 23, and 28 also harbored positional candidate genes (0.5 MB) near peak SNPs. The genes near peak SNPs were *ESR2*, *ITPR1*, *GNG2, RGS9BP, ANKRD27*, *TDRD12*, *GRM1, MTHFD1, PTGDR* and *NTNG1.* Among all positional candidate genes, some are well known as fertility-related genes. We listed selected candidate genes, based on current knowledge of reproductive biology (Table 3). For the full list of positional candidate genes, see Table S3.

Gene ontology and pathway analyses of the positional candidate gene list resulted in classification of the genes into 167 KEGG biological pathways (number of genes in pathway ≥ 6) (Table S4). However, none of the classified pathways were significantly enriched for functional over-representation when the default *Bos taurus* background gene list was used in the enrichment analysis in DAVID. Similarly, no biological process gene ontology terms were enriched for the target candidate gene list, when compared to the default background. However, using DAVID, we did classify the genes in the positional candidate list into several biological process, including *Transcription-DNA template*, *Translation*, *Cell proliferation*, and *spermatogenesis* (Table S4).

When the positional candidate gene list was analyzed for gene ontology terms and pathways enrichment using the trained background gene list, 66 KEGG biological pathways were significantly enriched (Benjamini corrected *p*-value < 4.6 × 10^−2^) (Table S4). Selected biological pathways, which are directly related to fertility, as per prior literature-based knowledge, are listed in Table 4 (they are a subset of Table S4).

**Figure 2:**
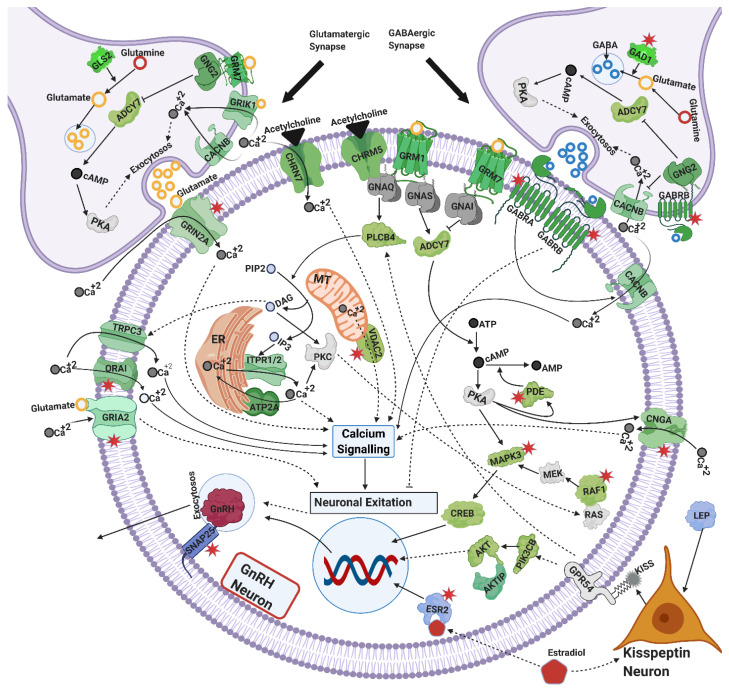
Mechanisms of GnRH secretion in the hypothalamus: insights from positional candidate genes identified in the meta-analyses of heifer fertility traits. Figure includes interaction of neurotransmitters from glutamatergic, GABAergic, cholinergic and Kisspeptin neurons with their respective receptors to excite GnRH neurons through calcium and cAMP signaling for GnRH secretion. Proteins in green represent the positional candidate genes, within 0.5 Mb of significant SNPs, which are also known in these KEGG pathways. Blue proteins represent the positional candidate genes placed in these intricate mechanisms based on the literature (but are not known from KEGG pathways). Grey proteins were not represented in the positional candidate gene list, but are components of the proposed mechanism due to current literature [6,10,47,48,49,50,51,52,53,54,55,56,57,58,59,60,61,62,63,64,65,66,67] and KEGG pathways. Red stars identify proteins from this study in common with previous proteomics and transcriptomics analyses of pre- vs post-pubertal heifers.

**Figure 3:**
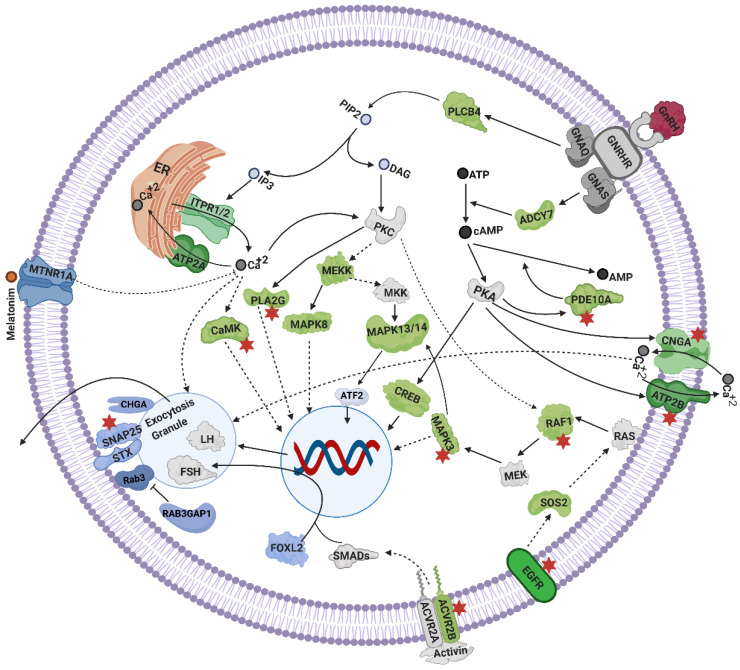
Mechanisms of gonadotrophins secretion in the pituitary gland: insights from positional candidate genes identified in the meta-analyses of heifer fertility traits. GnRH after binding to its receptor activates multiple signaling pathways including calcium, cAMP and growth factor signaling to express and secrete FSH and LH. Proteins in green represent positional candidate genes (within 0.5 Mb of significant SNPs), which were also reported in respective KEGG pathways. Blue proteins represent positional candidate genes integrated here because of current literature cited (but are not known from KEGG pathways). Grey proteins indicate the genes which were not listed as positional candidates, but are components of the proposed mechanisms due to known pathways or current literature [14,67,68,69,70,71,72,73,74,75,76,77]. Red stars identify proteins from this study which are in common with previous proteomics and transcriptomics analyses of pre- vs post-pubertal heifers.

**Figure 4:**
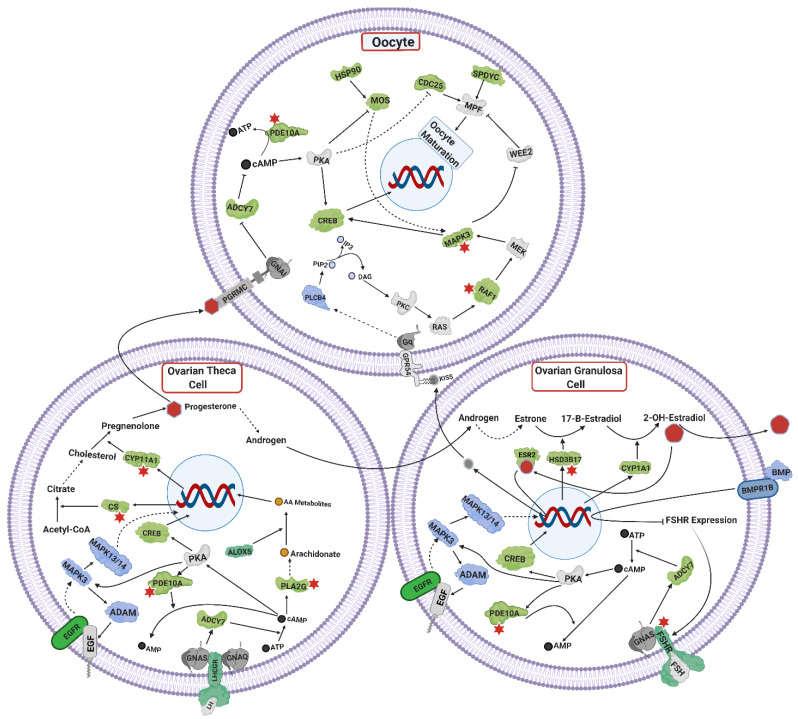
Mechanisms of steroid synthesis and oocyte maturation in ovarian cells: insights from positional candidate genes identified in the meta-analyses of heifer fertility traits. Receptors for LH and FSH in theca and granulosa cells initiate the signaling cascades for steroidogenesis, follicular growth and oocyte maturation. Proteins in green represent positional candidate genes (within 0.5 Mb of significant SNPs) for which there is also evidence from KEGG pathways. Blue proteins represent positional candidate genes placed in the proposed mechanisms because of current literature (but are not identified in KEGG pathways). Gray proteins indicate the genes that were not positional candidates, but were included based on current literature [67,78,79,80,81,82,83,84,85,86,87,88,89]. Red stars mark genes were in common between this study and previous proteomics and transcriptomics analyses of pre- vs post-pubertal heifers.

Our functional annotation allowed grouping of position candidate genes according to their biological role. The MAPK signaling pathway, which includes kinases and regulates proteins in a variety of biological pathways, was represented by 32 genes in the positional candidate gene list. Out of these, nine were protein kinases, including *MAPK3, MAP3K20, MAP3K19, MAPK14, MAPK13, MAPK8, MAPK6, MAP3K1* and *MAP3K11*, and were within 0.5 Mb of significant SNPs on chromosomes 2, 10, 23, 25, 28, and 29. Two growth factors, bone morphogenic protein−5 (*BMP5*) on chromosome 23 and platelet-derived growth factor-C (*PDGFC*) on chromosome 17, were also near significant SNPs. Receptors of different growth factors, such as *BMPR1B, PDGFRA, PDGFRB* and *EGFR* and the receptor of the growth hormone *GHR,* were in the positional candidate gene list close to significant SNPs on chromosomes 6, 7, 20 and 22. The positional candidate gene list also included cytokines and cytokine receptors—*IL26, IL7, IL19, IL20, IL24, IL16, IL27, IFNG, IL1R1, IL1RL2, IL1RL1, IL18R1* and *IL19RAP*—and two adipokines, *LEP* and *APLN.* Five genes including *GABRG1, GABRA2, GABRA4* and *GABRB1* belonged to GABAergic Synapse. Seven polymerase genes including *POLR3B, POLN, POLE2, PRIMPOL, POLR3A, POLA1 and POLA2* were mapped within the 0.5 Mb region of significant SNPs in this study. In total, 86 olfactory receptors were nearby significant SNPs and were included in our positional candidate gene list. Classification of positional candidate genes into biological pathways and functional groups allowed curation of integrated pathways and molecular mechanisms that may contribute to pubertal development and therefore impact on heifer fertility (Figure 2, Figure 3 and Figure 4).

The functional roles of positional candidate genes identified in this study were ascertained from the previous literature, and known biological pathways. With the help of previous literature, the positional candidate genes related to glutamatergic, GABAergic, and cholinergic synapses, were integrated into molecular mechanisms that contribute to GnRH secretion (Figure 2), and therefore puberty. Further, positional candidate genes involved in calcium, cAMP, estrogen, MAPK, PIK3B, and adipocytokines signaling, were identified as part of the mechanisms that excite GnRH neurons, prior to GnRH secretion by the exocytosis machinery. In summary, functional annotation of the positional candidate genes led to an integrated view of hypothalamic mechanisms that regulate in GnRH secretion (Figure 2).

The functional annotation of positional candidate genes also led to insights about the signaling mechanisms in pituitary gland that lead to gonadotrophin synthesis and secretion. The positional candidate genes PLA2G, CaMK, MAPK3, RAF1, PDE10A, ATP2B, CNGA, EGFR, and ACVR2B could be seen as part of the complex mechanisms of pituitary signaling (Figure 3), and they were also reported in previous proteomics and transcriptomics studies related to heifer puberty. These and more positional candidate genes fit with GnRH, calcium, cAMP, activin, melatonin, and MAPK signaling mechanisms that are important for the synthesis and secretion of gonadotrophins from the pituitary gland (Figure 3). Together, functions of positional candidate genes and current knowledge of pathways formed an integrated view of the mechanisms that may influence FSH and LH secretion (Figure 3), important for female reproduction.

Functional annotation of positional candidate genes led to propose an integrated mechanism of steroids synthesis and oocyte maturation, in ovarian tissue. Positional candidate genes coded for proteins involved in ovarian steroidogenesis, progesterone mediated oocyte maturation, cAMP signaling, PIK3B signaling and MAPK signaling (Figure 4). The integration of positional candidate genes across these known pathways led to the illustration of molecular mechanisms within theca and granulosa cells, as well as the oocyte, giving an overview of ovarian function (Figure 4).

Positional candidate genes were cross-checked with lists of differentially expressed genes and proteins from previous studies on heifer puberty and 392 genes in common were identified. Notably, fertility-related genes were among the 392 in genes common: *CS, BRAF, GABRA2, GABR1B, GAD1, FSHR, CNGA3, PDE10A, SNAP25, ESR2, GRIA2, ORAI1, EGFR, CHRNA5, VDAC2, ACVR2B, ORAI3, CYP11A1, GRIN2A, ATP2B3, CAMK2A, PLA2G, CAMK2D, HSD17B* and *MAPK3*. Among these common genes *BRAF, GABRA2, GABR1B, CNGA3, PDE10A, SNAP25, ESR2, GRIA2, ORAI1, VDAC2, ORAI3, GRIN2A* and *MAPK3* are part of the mechanisms involved in GnRH secretion (Figure 2). Among these common genes, some were considered part of gonadotrophin secretion mechanisms in the pituitary gland (*BRAF, CAMK2A, CAMK2D, SNPA25, ACVR2B, PDE10A, EGFR* and *PLA2G*, see Figure 3), while others were annotated as part of the ovarian pathways (*CS, FSHR, HSD17B, PDE10A, BRAF, MAPK3,* and *CYP11A1*, see Figure 4).

The positional candidate gene list included 133 transcription factors, 40 transcription co-factors and 15 chromatin-remodeling factors. Three transcription factors, *TAL2, ESR2* and *ZBTB1,* were nearby highly significant SNPs (*p*-value < 9 × 10^−9^). The positional candidate gene list included 48 transcription factors possessing zinc finger domains. *PLAG1*, a zinc finger-containing transcription factor, is located within 0.5 Mb of BovineHD1400007251 (*p*-value = 1.7 × 10^−7^)*,* and two more significant SNPs on chromosome 14. In addition, we used the trained background gene list to identify transcription factors that might be related to heifer fertility. The transcription factor AP2-α (*TFAP2A*) was the top regulator with the highest affinity score 14.38 (*p*-value ~ 0) for the trained background gene list. *TFAP2A* is located on chromosome 23, in the 0.5 Mb window of BovineHD2300013198, a peak SNP (*p*-value = 1.7 × 10^−6^). Two more transcription factors of this same class, *TFAP2B* and *TFAP2D,* were in the vicinity of BovineHD2300006011 (*p*-value = 7.6 × 10^−6^) on chromosome 23.

## 4. Discussion

Heritabilities of age at first calving and rebreeding score reported for *Bos indicus* cattle in Brazil are 0.10 and 0.22, respectively [90]. Heritability of PREG1 after fixed time artificial insemination is recorded as 0.18 in a different population of Brahman heifers [16]. We found heritabilities of REB and PREG1 as 0.28 and 0.17, respectively, which are similar to the estimates in cited studies. Although there were no other studies on FCS, this newly defined trait is based on early conception in the mating season and as such resembles age at first calving or a refinement of PREG1. Heritability of FCS was estimated as 0.11 in our study, which is similar to the heritability estimates of 0.10 for age at first calving in Brahman cattle in Brazil [90]. Three traits in this study were based on the similar records that confirmed pregnancy (or failure) after the first two mating seasons. Heifers can conceive in the first two mating seasons only after they have achieved puberty, marked as the first ovulation, which can be followed by the observation of the first *corpus luteum* [2]. Therefore, PREG1, FCS, and REB are all influenced by the same biological phenomenon: puberty. The same biological basis of the three traits confirmed high genetic correlations among these traits which was estimated in this study. In this study, after estimating heritabilities, we performed individual trait GWAS followed by meta-analysis.

Association studies, such as GWAS carried out on single traits that have low heritability expect SNP association with low-to-moderate significance, resulting in a high FDR [25]. Meta-analysis across traits may mitigate this problem and help to identify significant SNPs [23]. In the meta-analysis, we combined PREG1, FCS, and REB information that identified 1359 SNPs with a *p*-value lower than 9.0 × 10^−6^ and FDR < 0.0001, providing evidence for SNP associations that were not clear in the single-trait GWAS. Significant SNPs served as landmarks to propose positional candidate genes, which were subjected to pathway enrichment analyses and functional annotation. As a result, positional candidate genes were implicated in intricate mechanisms that influence puberty and fertility: pathways that trigger GnRH secretion in the Hypothalamus, mechanisms related to gonadotrophin secretion in the pituitary, and steroidogenesis as well as oocyte maturation in the ovaries.

Among positional candidate genes, 392 genes had been associated with heifer puberty before, in either transcriptomics or proteomics analyses of another set of Brahman cows. These genes identified in other “omics” studies add evidence to the role of these genes in the proposed mechanisms in this study. To facilitate the discussion, positional candidate genes were grouped into known pathways. Yet, first, we highlight genes of interest located in the vicinity of peak SNPs.

### 4.1. Genes Located within 0.5 Mb of Peak SNPs

We considered every SNP with a *p*-value < 10 × 10^−9^ as a peak SNP. The 0.5 Mb genomic region around a peak SNP on chromosome 8 contains six genes *SLC44A1, FSD1L, FKTN, TAL2* and *TMEM38B*. The same genomic region, but on chromosome 9 in humans, including the homologous genes *FKTN, FSD1l, TAL2* and *TMEM38B*, is associated with age of female at sexual maturation [91]. Two more GWAS reported *SLC44A1* and *TMEM38B* as candidate genes for age at first sex and pubertal growth in humans [92,93].

The peak SNP region on chromosome 18 harbored the genes *RGS9BP, ANKRD27* and *TDRD12*. Variants in vicinity to this region are associated with daughter pregnancy rate in Holstein cattle [94]. A peak SNP in chromosome 3 pointed to *VAV3* as a candidate gene in this study. An SNP near this gene is associated with “interval from first to last insemination”, a trait that contributes to the fertility index in Nordic Red cattle [95]. The gene *IDH3A* was in the peak SNP region on chromosome 21. The protein coded by *IDH3A* was up regulated in the ovaries of post-pubertal heifers as compared to pre-pubertal heifers [96]. The gene *PTGDR* was within a peak SNP region in this study and had been reported as a candidate in a previous GWAS as associated with age at first calving in cattle [97]. In short, these candidate genes suggested by peak SNPs have supporting evidence from previous studies. Nonetheless, their role in heifer fertility requires further validation, because assigning genes to significant SNPs purely on genomic location is imperfect. For example, the peak SNP in chromosome 3 could point to *VAV3* as discussed above, or it could suggest the candidate gene *NTNG1* that is involved with axon guidance and can disturb glutamatergic and dopaminergic signaling [98]. The axon guidance pathway coordinates migration of neurons and their connectivity, which influences GnRH neurons and therefore puberty. More about the role GnRH and glutamatergic neurons in puberty is described in the following sections.

The gene *ESR2* is another positional candidate, suggested by the peak SNP in chromosome 10, which seems obvious from a biological perspective. Estrogen receptors, like *ESR2*, are crucial in the reproductive axis [99,100]. A mutation in *ESR2* resulted in a lack of puberty and complete failure of the ovarian function in women [101]. In mice, a mutated *ESR2* can cause infertility [102]. Still, one cannot jump to conclusions, because the same peak SNP could suggest another gene: *MTHFD1*. It is less obvious, but *MTHFD1* is involved in DNA methylation, and its mutations can cause neuronal abnormalities [103] that may also affect puberty.

### 4.2. Exocytosis and GnRH Secretion

The hormone GnRH is considered the gatekeeper of puberty in cattle and other mammals. The secretion of GnRH is a complex phenomenon that requires coordinated molecular mechanisms in multiple neurons in the hypothalamus. The neuron secretion of GnRH and other neurotransmitters is dependent on the exocytosis machinery. Proteins such as synapsins, tropomysins, synaptosomal-associated proteins, syntexins, synaptogamins, and liprins are involved in the secretion of GnRH, gonadotrophins and neurotransmitters from their secretory vesicles [77,104,105]. The positional candidate gene list included such proteins: *SYN3, TPM1, SNAP25, STX2, STX4, STX11, STX19, STX1B, SYT11* and *PPFIA2*. Calcium and cAMP signaling coordinate with these proteins in the exocytosis of GnRH, gonadotrophins and neurotransmitters at large [65,77,104,105]. Neurotransmitters secreted from neighboring neurons interact with GnRH neurons to trigger GnRH secretion. Neurotransmitters such as kisspeptin, glutamate, GABA, acetylcholine and melatonin bind to their receptors and stimulate GnRH secretion through calcium and cAMP signaling [10,11,12,64,106,107,108,109]. These mechanisms that contribute to exocytosis in neurons and to GnRH secretion were compiled in Figure 2, where the implicated candidate genes are represented in green. The following discussion expands on all the mechanisms illustrated in Figure 2, Figure 3 and Figure 4.

#### 4.2.1. Calcium Signaling

Twenty-six positional candidate genes were part of the calcium signaling pathway in this study. To name a few, *ITPR1, ITPR2, TRPC3, CACNB2, VDAC2, ORA11, ORA13* and *PLCB4*, contribute to maintain calcium (Ca^++^) levels in cells [110]. High Ca^++^ levels are correlated with excitation and firing of GnRH neurons, which result in secretion of GnRH [111]. High Ca^++^ levels in GnRH neurons are achieved through two mechanisms including, the voltage-dependent Ca^++^ influx from extracellular resources and the secretion of Ca^++^ from intracellular calcium [112]. The gene *CACNB2* encodes the β subunit of L-type calcium receptors. L-type calcium receptors mediate the effects of GABA and glutamate signaling to regulate calcium intake from extracellular resources into GnRH neurons [113]. Kisspeptin signaling, via *GPR54* and *PLCB4*, regulates Ca^++^ levels in GnRH neurons through *TRPC*3 channels from extracellular sources, and through *ITPR1/2* from intracellular sources [47,111,114,115]. Additionally, *TRPC* channels coordinate with *ORAI* calcium channels to modulate Ca^++^ levels in hypothalamic neurons [116,117]. The gene *VDAC2* also regulates calcium levels from intracellular sources [118]. In short, these positional candidate genes linked to calcium signaling might contribute to the studied phenotypes of heifer fertility because they fit with the mechanisms of GnRH secretion via calcium signaling.

#### 4.2.2. Cyclic AMP (cAMP) Signaling

Cyclic AMP (cAMP) is an important secondary messenger in the regulation of GnRH secretion. The positional candidate gene list included 25 genes of the cAMP signaling pathway in this study. Among these, *ADORA2A, ADCY7, CNGA1, CNGA3,* and four phosphodiesterases (*PDE4A, PDE6A, PDE10A* and *PDA11A*) are linked to cAMP signaling. Provision of cAMP increases GnRH secretion in GT1-7 neurons [119]. Studies report that the cAMP regulated *CNG* channels cause depolarization of GTI-7 neurons by increased Ca^++^ levels which results in GnRH secretion [64,120]. Phosphodiestrases hydrolyze cAMP and are implicated in decreasing GnRH secretion from GTI cells by decreasing cAMP levels [63,120]. *ADORA2A* is involved in *ADCY7* activation for cAMP production [121]. In GnRH neurons, *ADORA2A* is differentially expressed in pro-estrous and meta-estrous of mice, playing a role in the estrous cycle [122]. The positional candidate genes related to cAMP signaling might impact on heifer fertility because these mechanisms contribute to GnRH and gonadotrophin secretion, which are key hormones for reproductive function.

#### 4.2.3. Glutamatergic and GABAergic Synapse

Sixteen genes from the positional candidate gene list were linked to glutamatergic synapse. Among these genes, *GNG2, GRIA2, GRIN2A, GRM1, GRM7, ITPR1,* and *ITPR2* operate by regulation of Ca^++^ signaling in GnRH neurons [6]. In Iranian Holstein cattle, a polymorphism near *GRIN2A* is associated with age at first calving [123]. The glutamate neurotransmitter activates *GRIA2* (*AMPA*) and *GRIN2A* (*NMDA*) receptors [6] which further regulate Ca^++^ in GnRH neurons and cause secretion of GnRH [124]. Glutamate signaling through *GRM7*, *GNG2* and *ADCY7* regulate auto-inhibition of glutamate secretion in the pre-synaptic membrane [125]. In the post-synaptic membrane in GnRH neurons, *GRM1* is involved in GnRH secretion through cAMP signaling [126,127]. In short, these genes involved with glutamatergic synapses excite GnRH neurons.

While glutamatergic synapses are excitatory, GABAergic synapses result in inhibitory effects on GnRH neurons [108]. Four GABAergic receptors (*GABRA4, GABRA2, GABRB1* and *GABRG1*) were identified in positional candidate gene list. Signaling of GABA through *GABRA* receptors coordinates with *NMDA* and *AMPA* receptors to activate L-type Ca^++^ channels (*CACNB2,*) to elevate Ca^++^ levels for depolarization that results in GnRH secretion [112]. The same study describes the inhibitory effects of the receptor *GABRB* on excitation of GnRH neurons [113]. Importantly, during puberty the effect of GABA signaling through its *GABRA* and *GABRB* receptors inhibits secretion of GnRH [128]. The coordinated glutamatergic and GABAergic pathways impact on pubertal development as they affect GnRH signaling. After puberty, these mechanisms (and by extension the identified genes) continue to be important for reproductive function as they orchestrate hypothalamic activity.

#### 4.2.4. Cholinergic Synapse

Seventeen genes from the positional candidate gene list are known for their role in cholinergic synapse. Out of these, five were nicotinic acetylcholine receptors (*CHRNA3, CHRNA5, CHRNA7*, *CHRNA9* and *CHRNB4*). Nicotine and acetylcholine through these receptors, especially *CHRNA7*, activate GT1-7 hypothalamic neurons and cause secretion of GnRH by elevating Ca^++^ levels [10]. *CHRNA7* is also believed to act as a calcium permeable channel which allows an influx of extracellular calcium into GnRH neurons stimulating hormonal secretion [10]. The muscarinic cholinergic receptor *CHRM5* couples with α subunit of G_q/11_ protein and activates phospholipase-C to elevate Ca^++^ levels for GnRH secretion [61]. Candidate genes linked to cholinergic synapse were integrated in the mechanisms that contribute to GnRH secretion (Figure 2), and therefore could be related to female reproduction.

#### 4.2.5. Melatonin Signaling

A melatonin receptor *MTNR1A* was identified in the positional candidate gene list. Melatonin may act as a regulator of the estrous cycle in seasonal breeders [129,130]. Melatonin, through *MTNR1A* also inhibits calcium and cAMP levels, which might result in suppression of LH release from the pituitary gland (Figure 3). Melatonin signaling might influence the timing of puberty in seasonal breeders [76] and tropical heifers.

#### 4.2.6. Estrogen Signaling

Estrogen signaling is important at all levels of the reproductive axis, in the hypothalamus, the pituitary gland and the ovaries. The genes *ESR2, PIK3CB, AKT1, ITPR1, ITPR2, CREB3L2* and *MAPK3* are members of estrogen signaling pathways and were identified as positional candidates in this study. These genes are involved in estrogen signaling that mediates GnRH secretion [131,132]. In GnRH neurons, *ESR2* is expressed where it imparts rapid gene regulatory actions by phosphorylating *CREB* [133]. In GT-17 (GnRH producing) neurons *ESR2* in the presence of estradiol increases cAMP production, activates calcium signaling, and causes GnRH secretion in a dose-dependent manner [134]. Estrogen signaling also works through kisspeptin neurons and regulates secretion of GnRH in hypothalamus [135]. The kisspeptin neurons in the anteroventral periventicular (AVPV) nucleus of the hypothalamus are believed to mediate positive feedback of estrogen signaling [136]. Kisspeptin neurons in the AVPV nucleus of the hypothalamus express high levels of estrogen receptor ESR2 [137]. It is not clear yet, but *ESR2* along with ESR1 is implicated in regulation of GnRH through KISS signaling from AVPV nucleus of hypothalamus [138]. Leptin is implicated with the onset of puberty and related disorders in human females [139]. Leptin is implicated with the onset of puberty and related disorders in human females [139]. Leptin also directly or indirectly regulates GnRH secretion through Kisspeptin neurons [140,141,142,143]. It is not difficult to propose that estrogen signaling genes might impact heifer fertility and therefore could be proposed as both positional and functional candidate genes.

### 4.3. Mechanisms Linked to Gonadotrophins Secretion by the Pituitary

GnRH via its receptor stimulates the pituitary gland to secrete LH and FSH which then control ovarian function in an endocrine manner. Calcium and cAMP signaling work in synergy for the exocytosis of LH from pituitary cells [13]. The genes *CREB5*, *PLCB, PLA2* (subunits G3, *G7, G6, G1B, G4B, G4E, G4D*) *EGFR, SOS2, BRAF, RAF1, MRAS, MAPK3* (*ERK*), *MAPK8 (JNK*), *MAP3K1* (*MEKK*), *MAPK13/14* (*p38MAPK*), and *ACVR2B* were among positional candidates and were annotated as part of GnRH signaling cascades in the pituitary (Figure 3). This set of genes mediates the secretion of LH and FSH through *PKC* and *EGFR* signaling, involving MEKK, ERK and JNK signaling cascades [70,74]. Cyclic AMP also plays its role in the pituitary circuits through *PKA, MAPK* and *CREB* signaling [70]. EGFR through RAS, MAPK and p38MAPK signaling activates *ATF2* transcription factor, which along with c-*Jun* and *AlK1,* contributes to the transcription of α and β subunits of gonadotrophin genes [70]. Through ERK signaling, focal adhesion and actin cytoskeleton remodeling are involved in the expression of LH*β* [14]. Among positional candidates, 27 genes were part of the focal adhesion pathway while 25 were linked to actin cytoskeleton remodeling in this study. Activin receptor *ACVR2B* is known for its role in regulation of *FSHβ* transcription in gonadotrophs [75].

The positional candidate genes *CAMK1D, CAMK2D, CAMK2A* and *ATP2B3* are involved in calcium signaling mechanisms, which act on the pituitary for secretion of gonadotrophins. The gene *CAMK* through *ITPR1* and *ITPR2* mobilize Ca^++^ stores and facilitate the expression and secretion of LH and FSH [14,71,72,144]. Elevated Ca^++^ levels initiate rapid exocytosis of vesicles of gonadotrophins (LH and FSH) from pituitary cells [145]. Meanwhile, the role *ATP2B3* is clearance of Ca^++^ from pituitary cells afterwards [73].

Finally, some positional candidate genes could be linked to FSH and LH exocytosis. Five Syntaxins, *CHAG*, *SNAP25* and *RAB3GAP1* could be associated with exocytosis of gonadotrophins. Before exocytosis, FSH appears in granules associated with chromogranin-A (*CHGA*) [14]. The FSH and LH granules go through exocytosis with the help of exocytosis machinery including *SNAP25/SNARE/Rab3* and syntaxins [14,77]. Active *Rab3* is part of the exocytosis machinery and it is converted to the inactive form by an enzyme coded by *RAB3GAP1* [146].

### 4.4. Ovarian Steroidogenesis, and TGF Signaling

The positional candidate genes *FSHR, LHCGR, CYP11A1, CYP1A1, HSD17B, CREB* and *CS* are annotated to the ovarian steroidogenesis mechanisms (Figure 4). These genes are implicated in estrogen and progesterone synthesis from theca and granulosa cells of ovaries through FSHR and LHCGR signaling [78]. The cascade of FSHR/LHCGR and *PKA*, activates *CREB* which acts as a transcription factor for synthesis of steroid hormones in ovarian cells [147,148,149]. In Holstein cattle, an SNP near *FSHR* was associated with heifer conception rate, while another SNP linked to *LHCGR* gene is associated with the productive life of cows [150]. In Chinese Holstein cows, an SNP in the 5′ UTR region of *FSHR* is associated with superovulation [151]. The gene *CS* converts acetyl-CoA to citrate [152] which contributes to steroid synthesis downstream [96]. Steroidogenic genes CYP11A and HSD17B catalyze reactions of conversion of cholesterol to pregnanolone and estradiol-1 to estradiol-2, respectively [78].

The positional candidate genes *BMP5, BMP8A*, *BMPR1B, GDF2,* and *GDF10* are part of TGF-β superfamily. Bone morphogenic proteins (BMPs) and growth differentiation factors (GDFs) are implicated in ovarian steroidogenesis [78]. Bone morphogenic proteins (BMPs) and their receptors, are important regulators of follicular growth and androgen production in granulosa and theca cells [153,154]. Through their receptor *BMPR1B*, BMPs negatively impact the effect of FSH on ovarian cells by reducing the expression of its receptor *FSHR* [155]. Mutation in *BMPR1B* in sheep is associated with increased ovulation rate [156].

Multiple interleukins were identified as part of the positional candidate gene list in this study. Cytokines do interfere with steroidogenesis in a systemic manner [157]. Interleukin receptor *IL1R1* was among the mapped genes in this study. The *IL-1* through its receptor *IL1R1,* is involved in proliferation of theca and granulosa cells, and regulates steroidogenesis in mammals [78,158].

### 4.5. Oocyte Maturation

Oocyte maturation is crucial to cattle fertility [159]. Incompetent oocytes are a major reason for reduced fertility [160]. A complex interplay happens between steroids of theca cells, granulosa cells, and the oocyte for its maturation to occur. Positional candidate genes such as *MAPK3, ADAMs, EGFR, MAPK13/14, PDE10A, CDC25A, ADCY7, SPEDY, MAPK8, ESR2, PLCB, MOS, KIT* and *PABPC* were all integrated in the oocyte maturation mechanisms (Figure 3). LH signaling activates *MAPK3* signaling which induces metalloproteinases (*ADAMs)* in granulosa cells. Epidermal growth factor (*EGF)* cleaved by *ADAM* activates its receptor *EGFR*. The *EGFR* in turn through *RAS, MAPK3* pathway activates *MAPK13/14* [161,162] which further activates transcription factors involved in oocyte maturation in pigs [163,164]. The *EGF* through *EGFR* also regulates synthesis of progesterone from theca cells and estrogen in granulosa cells. The progesterone through its membrane receptors is involved in oocyte maturation in mammals [165]. *ADCY7, SPDYC* and *MAPK8*, are also implicated in progesterone-mediated oocyte maturation [85,166,167]. Till now, the exact mechanisms are unknown, but somehow LH, EGFR, KISSR and progesterone signaling coordinate and play a vital role in mammalian oocyte maturation [168,169,170,171].

The gene *PDE10A* is expressed in granulosa cells and oocytes [172]. Increased cAMP levels activate *PDE10A* which after activation hydrolyses cAMP and cGMP to decrease their levels [173]. The decreased level of cAMP inactivates PKA and as a result *CDC25A* is activated. Activated *CDC25* activates the meiosis promoting factor (MPF) which causes oocyte maturation [174].

Another recently reported mechanism of oocyte maturation involves estrogen and kisspeptin signaling in ovaries. The *ESR2* receptor in granulosa cells, after receiving estradiol, promotes *KISS* transcription. Paracrine action of *KISS* from granulosa cells activates *KISSR* in the oocyte, which further regulates oocyte maturation through *PLCB, DAG, PKC, RAS* and *MAPK3* in a signaling cascade, in rats [171]. This same study in rats, observed the upregulation of *MOS, CDC25A, KIT* and *PABPC* in oocytes after treatment with *KISS* agonist. These genes are also positional candidate genes in our meta-analysis. The gene *MOS* is one of the initially translated maternal mRNAs in oocyte, which further stimulates the oocyte maturation process [175]. The genes *KIT* and *PABPC* improve oocyte maturation by overcoming inhibitory effects of natriuretic peptide C *(NPPC)* signaling, and improving the stability of mRNAs for translation, respectively [176,177].

### 4.6. Olfactory Signaling

We identified 86 genes from the positional candidate gene list as olfactory receptors. Olfactory receptors are expressed in olfactory receptor neurons and are involved in smell signal transduction. Pheromones present in bull urine contribute to onset of puberty in beef heifers [178]. Similarly, estrous pheromones can help bulls to detect pubertal heifers for mating [179]. Mating behavior is dependent on the olfactory receptors on vomeronasal olfactory neurons (VNO) and the main olfactory epithelium (MOE). Olfactory receptors transmit the signals of pheromones through VNO and MOE. These olfactory apparatuses extend their neuronal projections near the medial preoptic area (MPOA) region of hypothalamus where GnRH neurons reside [180]. Thus, chemo signaling for mating behavior is coordinated through GnRH neurons. Chemical disruption of VNO and MOE olfactory apparatuses impairs mating behavior and reduces *MAKP* phosphorylation in GnRH neurons [180]. Olfactory receptors were associated with reproductive traits herein and previously, in Nellore cattle [181,182].

### 4.7. Transcription Factors

The positional candidate gene list included three genes of the TFAP2 transcription factor family *TFAP2A, TFAP2B, TFAP2D* and 36 zinc finger transcription factors, one of which is *PLAG1*. Analysis of the fertility related trained background gene list in this study resulted in the identification of *TFAP2A* as a top regulator of reproduction-related genes. The family of TFAP2, especially *TFAP2A* and *TFAP2B*, are key regulators of gene expression in the bovine placenta and contribute to gestation progression [183]. TFAP2 is continuously required to produce LHRH in the forebrain of developing mice [184]. The *PLAG1* gene has been associated with reproductive precocity in cattle in multiple studies [24,185].

Other transcription factors identified in the positional candidate gene list were *FOXL2I, TTF1, POU5F1, NFAT5,* *PHTF1* and *HDAC4.* The *FOXL2I* is important for female reproduction in endometrium, ovarian and hypothalamic–pituitary tissues [186]. Transcription factor *TTF1* binds to the promoter sequence of GnRH and regulates transcriptional expression of GnRH in rats [187]. Transcription factor *POU5F1* is important for oocytes, zygotes and embryos. For example, it is crucial for blastocyst formation in bovine embryos [188]. Transcription factor *NFAT5* is differentially expressed in the hypothalamus of post-pubertal as compared to pre-pubertal Brahman cows [43] and SNPs associated with this gene are also associated with age at menarche in humans [189]. The gene *PHTF1* is associated with fertility index in Holstein cattle [190]. The genes *HDAC4*, a histone deacetylase, is reported to remodulate the male chromatin within oocytes to proceed for development of normal zygotes after fertilization [191]. In short, the role of these transcriptions factors in heifer fertility should be further researched.

## 5. Conclusions

The multi-trait meta-analysis allowed us to identify significant SNPs and propose positional candidate genes. Mining the list of candidate genes for biological function led to an integrated view of signaling mechanisms in the hypothalamus, pituitary and ovarian tissues that are of known importance for fertility and might contribute to the studied heifer traits. Further validation of the identified SNPs is required.

## Figures and Tables

**Table 1:** Scoring criteria of early reproductive traits in Brahman heifers.

No.	Trait	Score	Scoring Criteria
1	PREG1	1	Not pregnant as a result of the first mating opportunity (*n* = 600)
		2	Pregnant as a result of the first mating opportunity (*n* = 1719)
2	FCS	1	Never conceived up to 36 months of age (*n* = 429)
		2	Conceived between 29 and 36 months of age (*n* = 436)
		3	Conceived before 29 months of age (*n* = 1150)
3	REB	1	Not pregnant as a result of the first two mating opportunities (*n* = 153)
		2	Pregnant as a result of the second mating opportunity, but not the first (*n* = 550)
		2.5	Pregnant as a result of the first mating opportunity, not the second (*n* = 506)
		3.5	Pregnant twice, as a result of the first two mating opportunities (*n* = 326)

PREG1: Pregnancy outcome after first mating chance. FCS: first conception score. REB: rebreeding score.

**Table 2:** Heritabilities, genetic and phenotypic correlations for reproductive traits in Brahman cows.

Traits	PREG1	FCS	REB
PREG1	0.17 (0.03)	0.839 (0.06)	0.799 (0.07)
FCS	0.86 (0.01)	0.11 (0.03)	0.756 (0.1)
REB	0.73 (0.02)	0.65 (0.02)	0.28 (0.05)

Diagonal from top-left to bottom-right represents heritabilities of the traits. Above the diagonal are genetic correlations of the traits. Below the diagonal are phenotypic correlations of the traits. Standard errors are in parentheses. PREG1: Pregnancy outcome after first mating chance. FCS: first conception score. REB: rebreeding score.

**Table 3:** Genes known for their reproductive biology function, in the vicinity of significant SNPs.

SNP	Gene	BTA	Location of SNP	*p*-Value	Function	Overall SNP Effect
BovineHD1100009366	*LHCGR*	11	31339285	7.8 × 10^−6^	Steroid Synthesis	0.057
BovineHD1100009366	*FSHR*	11	31339285	7.8 × 10^−6^	Steroid Synthesis	0.057
BovineHD4100003128	*LEP*	4	92253894	4.0 × 10^−6^	GnRH Secretion	0.053
BovineHD1400007251	*MOS*	14	23304037	1.8 × 10^−7^	Oocyte Maturation	0.059
BovineHD2200014848	*CDC25A*	22	51689566	4.6 × 10^−6^	Oocyte Maturation	−0.050
BovineHD2200003516	*AVCR2B*	22	11918372	4.6 × 10^−7^	TGF-β Signaling	−0.056
BovineHD2200000211	*EGFR*	22	878627	8.0 × 10^−6^	GnRH Signaling	−0.052
BovineHD2500007459	*MAPK3*	25	26160282	3.4 × 10^−6^	GnRH Signaling	0.060
BovineHD1000021917	*ESR2*	10	76586616	3.0 × 10^−10^	Estrogen Signaling	−0.114
BovineHD0900023775	*GRM1*	9	83806867	9.5 × 10^−10^	Glutamate Signaling	−0.066
BovineHD1700011908	*GRIA2*	17	41973761	3.0 × 10^−6^	Glutamate Synapse	0.098
BovineHD2500002242	*GRIN2A*	25	8381736	4.2 × 10^−6^	Glutamate Synapse	0.057
BovineHD2200005404	*GRM7*	22	18702200	4.4 × 10^−6^	Glutamate Synapse	0.070
BovineHD0600018549	*GABRA4*	6	65504186	3.1 × 10^−6^	GABAergic Synapse	−0.050
BovineHD0200007364	*GAD1*	2	25614206	7.9 × 10^−6^	GABAergic Synapse	−0.053
BovineHD0600018549	*GABRB1*	6	65504186	3.1 × 10^−6^	GABAergic Synapse	−0.050
BovineHD0600018311	*GABRA2*	6	64738586	3.3 × 10^−6^	GABAergic Synapse	−0.048
BovineHD0600018311	*GABRG1*	6	64738586	3.3 × 10^−6^	GABAergic Synapse	−0.048
BovineHD1300000677	*PLCB4*	13	2565300	9.7 × 10^−6^	Calcium Signaling	0.047
BovineHD2200006328	*ITPR1*	22	21699681	5.2 × 10^−10^	Calcium Signaling	−0.092
BovineHD0400018696	*CREB5*	4	67587933	3.8 × 10^−6^	cAMP Signaling	−0.082
BovineHD1800005855	*ADCY7*	18	18675150	4.5 × 10^−7^	cAMP Signaling	0.059
BovineHD0600018878	*CNGA1*	6	66763069	7.2 × 10^−7^	cAMP Signaling	0.062
BovineHD0700033604	*PDE4A*	7	15081779	2.2 × 10^−7^	cAMP Signaling	0.064
BovineHD0300002075	*HSD17B7*	3	6617455	1.3 × 10^−6^	Steroid Synthesis	−0.052
BovineHD2100009894	*CYP11A*	21	34099081	4.2 × 10^−7^	Steroid Synthesis	−0.052
BovineHD1400007252	*PLAG1*	14	23313248	1.8 × 10^−7^	Transcription Regulation	0.059
BovineHD2300013198	*TFAP2A*	23	45590544	1.7 × 10^−6^	Transcription Regulation	0.058
BovineHD1100029888	*TTF1*	11	102587601	3.2 × 10^−6^	Transcription Regulation	0.046
BovineHD2100008703	*CHRNA7*	21	29677844	1.9 × 10^−7^	Cholinergic Synapse	−0.063
BovineHD2700004441	*MTNR1A*	27	16259785	1.1 × 10^−6^	Melatonin Receptor	−0.048

**Table 4:** Known reproductive pathways significantly enriched in the positional candidate gene list.

Pathway	Gene Count	Gene Names	Adj. *p*-Value *
GnRH Signaling	16	*RAF1, SOS2, ADCY7, CAMK2A, CAMK2D, EGFR, ITPR1, ITPR2, MAPK13, MAPK3, MAPK8, MAP3K1, PLA2G4D, PLA2G4E, PLA2G4B, PLCB4*	1.2 × 10^−3^
Progesterone Mediated Oocyte Maturation	13	*BRAF, RAF1, ADCY7, CDC25A, HSP90AA1, HSP90AB1, MAPK10, MAPK13, MAPK3, MAPK8, PIK3CB, SPDYC, MOS*	5.6 × 10^−3^
Estrogen Signaling	17	*FKBP5, RAF1, ADCY7, CREB3L2, ESR2, SOS2, GRM1, HSP90AA1, HSP90AB1, ITPR1, EGFR, ITPR2, MAPK3, OPRM1, PIK3CB, PLCB4, AKT1*	1.2 × 10^−3^
Glutamatergic Synapse	15	*GNG2, ADCY7, GRIA2, GRIN2A, GRIK1, GRM7, GRM1, GLS2, ITPR1, ITPR2, MAPK3, PLA2G4B, PLA2G4E, PLA2G4D, PLCB4*	7.0 × 10^−2^
Regulation of Actin Cytoskeleton	25	*BRAF, MRAS, LIMK2, RAF1, ARHGEF1, CHRM5, CYFIP2, ENAH, FGFR3, ITGA4, ITGA8, ITGAL, ITGB2, MAPK3, MYLPF, PAK2, PAK5, PIK3CB, PDGFC, PDGFRA, PDGFRB, MOS, VAV3, ITG5, ITGB2*	1.2 × 10^−3^
Cholinergic Synapse	17	*AKT1, GNG2, ADCY7, CREB3L2, CAMK2A, CAMK2D, CHRM5, CHRNA3, CHRNB4, CHRNA7, ITPR1, ITPR2, MAPK3, PIK3CB, PLCB4, SLC5A7, KCNQ4*	9.3 × 10^−4^
cAMP Signaling	24	*AKT1, ATP2B3, BRAF, FXYD1, RAF1, RAPGEF4, ADCY7, CREB3L2, CAMK2D, CNGA1, FSHR, GLP1R, GRIA2, GRIN2A, MAPK3, MAPK8, NPR1, OXTR, PIK3CB, PDE4A, VAV3, ORAI1, RELA, ADORA2A, CAMK2A.*	9.6 × 10^−4^
Calcium Signaling	26	*ATP2B3, ATP2A1, ORAI1, ORAI3, ADCY7, AGTR1, CAMK2A, CAMK2D, CHRM5, CHRNA7, EGFR, GRPR, GRIN2A, GRM1, ITPR1, ITPR2, ITPKA, LHCGR, OXTR, PLCB4, PLCD1, PHKG2, PDGFRA, PDGFRB, SLC25A4, VDAC2, ADORA2A*	3.6 × 10^−5^
Focal Adhesion	25	*AKT1, BRAF, RAF1, SOS2, CAPN2, COL4A3, COL4A5, COL4A6, ITGA4, ITGA8, ITGAB5, KDR, LAMC3, MAPK3, MAPK8, MYLPF, PAK2, PAK5, PIK3CB, PDGFC, PDGFRA, CAV3, PDGFRB, EGFR, VAV3, VWF, ZYX*	5.6 × 10^−4^
PI3K-Akt Signaling	40	*AKT1, CD19, GNG2, RBL2, RELA, RAF1, SOS2, CREB3L2, CASP9, CDC37, COL4A3, COL4A5, COL4A6, CSF1, CSF1R, CDK6, CDKN1A, EGFR, FGF19, FGFR3, GHR, HSP90AA1, HSP90AB1, ITGA4, ITGA8, ITGB5, IL7, KDR, LAMC3, LPAR1, LPAR3, MAPK3, PIK3CB, PDGFC, PDGFRA, PDGFRB, PPP2R5E, PPP2R2C, TSC1, VWF*	1.6 × 10^−3^
Ovarian Steroidogenesis	10	*HSD17B7, ADCY7, ALOX5, CYP11A1, CYP1A1, FSHR, LHCGR, PLA2G4B, PLA2G4D, PLA2G4E*	4.2 × 10^−3^

* Adj. *p*-value: *p*-value adjusted using FDR.

## Supplementary Materials

The following are available online at https://www.mdpi.com/article/10.3390/genes12050768/s1, Table S1: Distribution of animals for combined datasets of early reproductive traits. Table S2: Significant SNPs Resulting from Muti-Trait Meta-analysis of Three Early Reproductive Traits. Table S3: Genes Mapped within 0.5 MB Region of Significant SNPs; Representative Biological Pathways of the Genes; Transcription Factors; and Genes Common between Multi-Trait Meta-analysis and Transcriptomics and Proteomics. Table S4: DAVID Classified KEGG and GOTERM Biological Pathways.
