# Supplementary material for: Meta-Analysis of Heifer Traits Identified Reproductive Pathways in Bos indicus Cattle
Source: Genes (Basel). 2021 May 18;12(5):768. doi: 10.3390/genes12050768 (PMC8157873; doi:10.3390/genes12050768)
Supplement: Supplementary file 1 [file genes-12-00768-s001.zip › genes-1192850-supplementary/Supplimentary Table S1.pdf]

Table S1: Distribution of animals for combined datasets of early reproductive trait

No.	Traits	Dataset	Animals	Trait Score	Distribution of Animals
1	PREG-1	CRC	932	1	261
				2	671
		Kamilaroi	1208	1	211
				2	997
		NTDPI	179	1	128
				2	51
	**Total**	**3**	**2319**		**2319**
2	FCS	CRC	929	1	230
				2	41
				3	658
		Kamilaroi	1086	1	199
				2	395
				3	492
	**Total**	**2**	**2015**		**2015**
3	REB	CRC	932	1	26
				2	235
				2.5	387
				3.5	284
		Kamilaroi	424	1	116
				2	198
				2.5	103
				3.5	7
		NTDPI	179	1	11
				2	117
				2.5	16
				3.5	35
	**Total**	**3**	**1535**		**1535**
