# Supplementary material for: Meta-Analysis of Heifer Traits Identified Reproductive Pathways in Bos indicus Cattle
Source: Genes (Basel). 2021 May 18;12(5):768. doi: 10.3390/genes12050768 (PMC8157873; doi:10.3390/genes12050768)
Supplement: Supplementary file 1 [file genes-12-00768-s001.zip › genes-1192850-supplementary/Supplimentary Table S4.pdf]

Table S4: **<u>DAVID Classified KEGG Pathways and GOTERM Biological Processes</u>**

**Significantly enriched pathways using trained fertility related background gene list**				
Category	Term	Gene Count	P-Value	Benjamini
KEGG_PATHWAY	Calcium signaling pathway	26	1.90E-07	3.60E-05
KEGG_PATHWAY	cAMP signaling pathway	24	2.10E-06	1.80E-04
KEGG_PATHWAY	Rap1 signaling pathway	20	2.80E-06	1.80E-04
KEGG_PATHWAY	Focal adhesion	25	1.20E-05	5.60E-04
KEGG_PATHWAY	Ras signaling pathway	18	2.70E-05	9.30E-04
KEGG_PATHWAY	Cholinergic synapse	17	2.90E-05	9.30E-04
KEGG_PATHWAY	Neuroactive ligand-receptor interaction	17	4.30E-05	1.10E-03
KEGG_PATHWAY	MAPK signaling pathway	17	4.80E-05	1.10E-03
KEGG_PATHWAY	GnRH signaling pathway	16	6.00E-05	1.20E-03
KEGG_PATHWAY	Regulation of actin cytoskeleton	25	6.70E-05	1.20E-03
KEGG_PATHWAY	Estrogen signaling pathway	17	7.00E-05	1.20E-03
KEGG_PATHWAY	Tuberculosis	10	1.00E-04	1.60E-03
KEGG_PATHWAY	PI3K-Akt signaling pathway	40	1.10E-04	1.60E-03
KEGG_PATHWAY	Proteoglycans in cancer	16	1.30E-04	1.80E-03
KEGG_PATHWAY	Pathways in cancer	21	1.60E-04	2.10E-03
KEGG_PATHWAY	Prostate cancer	11	2.40E-04	2.90E-03
KEGG_PATHWAY	Choline metabolism in cancer	11	3.20E-04	3.60E-03
KEGG_PATHWAY	Ovarian steroidogenesis	10	4.10E-04	4.20E-03
KEGG_PATHWAY	Inflammatory mediator regulation of TRP channels	11	4.30E-04	4.20E-03
KEGG_PATHWAY	Alzheimer's disease	8	4.40E-04	4.20E-03
KEGG_PATHWAY	Aldosterone synthesis and secretion	10	5.60E-04	5.10E-03
KEGG_PATHWAY	NOD-like receptor signaling pathway	6	6.20E-04	5.40E-03
KEGG_PATHWAY	Progesterone-mediated oocyte maturation	13	6.70E-04	5.60E-03
KEGG_PATHWAY	Dopaminergic synapse	13	8.10E-04	5.90E-03
KEGG_PATHWAY	Platelet activation	13	8.10E-04	5.90E-03
KEGG_PATHWAY	Glioma	9	8.20E-04	5.90E-03
KEGG_PATHWAY	Retrograde endocannabinoid signaling	12	8.40E-04	5.90E-03
KEGG_PATHWAY	Central carbon metabolism in cancer	8	9.70E-04	6.60E-03
KEGG_PATHWAY	Glutamatergic synapse	15	1.10E-03	7.00E-03
KEGG_PATHWAY	cGMP-PKG signaling pathway	14	1.10E-03	7.00E-03
KEGG_PATHWAY	T cell receptor signaling pathway	9	1.50E-03	9.10E-03
KEGG_PATHWAY	ErbB signaling pathway	10	1.60E-03	9.50E-03
KEGG_PATHWAY	Glucagon signaling pathway	10	1.60E-03	9.50E-03
KEGG_PATHWAY	HTLV-I infection	12	1.70E-03	9.80E-03
KEGG_PATHWAY	Neurotrophin signaling pathway	10	1.80E-03	1.00E-02
KEGG_PATHWAY	Adrenergic signaling in cardiomyocytes	11	2.00E-03	1.10E-02
KEGG_PATHWAY	Vascular smooth muscle contraction	10	2.10E-03	1.10E-02
KEGG_PATHWAY	Fc epsilon RI signaling pathway	8	2.20E-03	1.10E-02

KEGG_PATHWAY	Metabolic pathways	11	2.30E-03	1.10E-02
KEGG_PATHWAY	Oxytocin signaling pathway	12	2.50E-03	1.20E-02
KEGG_PATHWAY	Hepatitis C	8	2.60E-03	1.20E-02
KEGG_PATHWAY	Long-term depression	8	2.60E-03	1.20E-02
KEGG_PATHWAY	Long-term potentiation	9	2.90E-03	1.30E-02
KEGG_PATHWAY	Natural killer cell mediated cytotoxicity	8	3.00E-03	1.30E-02
KEGG_PATHWAY	Leishmaniasis	6	3.00E-03	1.30E-02
KEGG_PATHWAY	TNF signaling pathway	8	3.90E-03	1.60E-02
KEGG_PATHWAY	Circadian entrainment	10	4.40E-03	1.80E-02
KEGG_PATHWAY	Prolactin signaling pathway	8	5.00E-03	2.00E-02
KEGG_PATHWAY	Gap junction	8	5.00E-03	2.00E-02
KEGG_PATHWAY	Huntington's disease	6	6.40E-03	2.40E-02
KEGG_PATHWAY	Melanoma	8	6.40E-03	2.40E-02
KEGG_PATHWAY	Amoebiasis	9	7.00E-03	2.60E-02
KEGG_PATHWAY	Toxoplasmosis	8	7.20E-03	2.60E-02
KEGG_PATHWAY	Pertussis	6	7.50E-03	2.70E-02
KEGG_PATHWAY	Pancreatic cancer	7	7.80E-03	2.70E-02
KEGG_PATHWAY	HIF-1 signaling pathway	8	8.00E-03	2.70E-02
KEGG_PATHWAY	Chagas disease (American trypanosomiasis)	8	1.00E-02	3.20E-02
KEGG_PATHWAY	Influenza A	7	1.00E-02	3.20E-02
KEGG_PATHWAY	Serotonergic synapse	11	1.00E-02	3.20E-02
KEGG_PATHWAY	Chemokine signaling pathway	10	1.10E-02	3.40E-02
KEGG_PATHWAY	Toll-like receptor signaling pathway	6	1.20E-02	3.70E-02
KEGG_PATHWAY	Signaling pathways regulating pluripotency of stem cells	8	1.20E-02	3.80E-02
KEGG_PATHWAY	RIG-I-like receptor signaling pathway	4	1.30E-02	3.90E-02
KEGG_PATHWAY	Epstein-Barr virus infection	6	1.40E-02	4.10E-02
KEGG_PATHWAY	Osteoclast differentiation	7	1.60E-02	4.60E-02
KEGG_PATHWAY	VEGF signaling pathway	7	1.60E-02	4.60E-02
**All classified KEGG biological pathways (Gene Count =>6) using default *Bos. taurus* background gene list of DAVID**				
KEGG_PATHWAY	Metabolic pathways	126	4.40E-01	1.00E+00
KEGG_PATHWAY	Olfactory transduction	86	9.90E-01	1.00E+00
KEGG_PATHWAY	Pathways in cancer	46	2.00E-01	9.00E-01
KEGG_PATHWAY	Neuroactive ligand-receptor interaction	42	1.40E-02	7.80E-01
KEGG_PATHWAY	PI3K-Akt signaling pathway	40	2.30E-01	9.70E-01
KEGG_PATHWAY	Ras signaling pathway	34	2.60E-02	7.80E-01
KEGG_PATHWAY	Rap1 signaling pathway	32	2.00E-02	7.80E-01
KEGG_PATHWAY	MAPK signaling pathway	32	1.40E-01	8.20E-01
KEGG_PATHWAY	Cytokine-cytokine receptor interaction	29	9.40E-02	7.80E-01
KEGG_PATHWAY	Chemokine signaling pathway	27	4.40E-02	7.80E-01
KEGG_PATHWAY	Focal adhesion	27	1.30E-01	8.00E-01
KEGG_PATHWAY	Calcium signaling pathway	26	8.40E-02	7.80E-01
KEGG_PATHWAY	Proteoglycans in cancer	26	1.50E-01	8.50E-01

KEGG_PATHWAY	Purine metabolism	25	6.80E-02	7.80E-01
KEGG_PATHWAY	cAMP signaling pathway	25	1.80E-01	8.70E-01
KEGG_PATHWAY	Regulation of actin cytoskeleton	25	2.80E-01	1.00E+00
KEGG_PATHWAY	Tuberculosis	24	1.30E-01	8.00E-01
KEGG_PATHWAY	Choline metabolism in cancer	22	6.00E-04	1.70E-01
KEGG_PATHWAY	Hepatitis C	21	3.90E-02	7.80E-01
KEGG_PATHWAY	Oxytocin signaling pathway	21	8.20E-02	7.80E-01
KEGG_PATHWAY	Influenza A	21	2.70E-01	1.00E+00
KEGG_PATHWAY	Viral carcinogenesis	21	8.00E-01	1.00E+00
KEGG_PATHWAY	Vascular smooth muscle contraction	20	2.50E-02	7.80E-01
KEGG_PATHWAY	Protein processing in endoplasmic reticulum	20	3.20E-01	1.00E+00
KEGG_PATHWAY	Biosynthesis of antibiotics	20	6.70E-01	1.00E+00
KEGG_PATHWAY	Endocytosis	19	9.30E-01	1.00E+00
KEGG_PATHWAY	HTLV-I infection	19	9.80E-01	1.00E+00
KEGG_PATHWAY	Platelet activation	18	1.30E-01	8.00E-01
KEGG_PATHWAY	Dopaminergic synapse	18	1.30E-01	8.00E-01
KEGG_PATHWAY	cGMP-PKG signaling pathway	18	4.10E-01	1.00E+00
KEGG_PATHWAY	Transcriptional misregulation in cancer	18	5.40E-01	1.00E+00
KEGG_PATHWAY	Estrogen signaling pathway	17	3.20E-02	7.80E-01
KEGG_PATHWAY	T cell receptor signaling pathway	17	5.50E-02	7.80E-01
KEGG_PATHWAY	Cholinergic synapse	17	7.70E-02	7.80E-01
KEGG_PATHWAY	Epstein-Barr virus infection	17	1.20E-01	8.00E-01
KEGG_PATHWAY	Neurotrophin signaling pathway	17	1.80E-01	8.70E-01
KEGG_PATHWAY	FoxO signaling pathway	17	2.40E-01	9.70E-01
KEGG_PATHWAY	Adrenergic signaling in cardiomyocytes	17	2.80E-01	1.00E+00
KEGG_PATHWAY	Jak-STAT signaling pathway	17	4.30E-01	1.00E+00
KEGG_PATHWAY	Cell adhesion molecules (CAMs)	17	4.70E-01	1.00E+00
KEGG_PATHWAY	GnRH signaling pathway	16	2.10E-02	7.80E-01
KEGG_PATHWAY	Prostate cancer	16	2.10E-02	7.80E-01
KEGG_PATHWAY	Gap junction	16	2.80E-02	7.80E-01
KEGG_PATHWAY	Pyrimidine metabolism	16	5.90E-02	7.80E-01
KEGG_PATHWAY	Inflammatory mediator regulation of TRP channels	16	9.00E-02	7.80E-01
KEGG_PATHWAY	Glutamatergic synapse	16	1.50E-01	8.50E-01
KEGG_PATHWAY	Leukocyte transendothelial migration	16	1.80E-01	8.70E-01
KEGG_PATHWAY	Axon guidance	16	2.60E-01	1.00E+00
KEGG_PATHWAY	Ribosome	16	3.50E-01	1.00E+00
KEGG_PATHWAY	Osteoclast differentiation	16	3.50E-01	1.00E+00
KEGG_PATHWAY	Hepatitis B	16	5.10E-01	1.00E+00
KEGG_PATHWAY	RNA transport	16	6.30E-01	1.00E+00
KEGG_PATHWAY	Alzheimer's disease	16	7.90E-01	1.00E+00
KEGG_PATHWAY	Herpes simplex infection	16	8.60E-01	1.00E+00
KEGG_PATHWAY	Glycerophospholipid metabolism	15	9.80E-02	7.80E-01
KEGG_PATHWAY	Phosphatidylinositol signaling system	15	1.00E-01	7.90E-01
KEGG_PATHWAY	TNF signaling pathway	15	1.80E-01	8.80E-01

KEGG_PATHWAY	Hippo signaling pathway	15	6.50E-01	1.00E+00
KEGG_PATHWAY	Huntington's disease	15	9.40E-01	1.00E+00
KEGG_PATHWAY	Long-term depression	14	4.50E-03	6.30E-01
KEGG_PATHWAY	ErbB signaling pathway	14	8.20E-02	7.80E-01
KEGG_PATHWAY	Pancreatic secretion	14	1.60E-01	8.50E-01
KEGG_PATHWAY	Serotonergic synapse	14	3.70E-01	1.00E+00
KEGG_PATHWAY	Sphingolipid signaling pathway	14	4.20E-01	1.00E+00
KEGG_PATHWAY	Insulin signaling pathway	14	6.00E-01	1.00E+00
KEGG_PATHWAY	Signaling pathways regulating pluripotency of stem cells	14	6.40E-01	1.00E+00
KEGG_PATHWAY	Alcoholism	14	9.90E-01	1.00E+00
KEGG_PATHWAY	Glioma	13	2.50E-02	7.80E-01
KEGG_PATHWAY	Progesterone-mediated oocyte maturation	13	1.60E-01	8.50E-01
KEGG_PATHWAY	Circadian entrainment	13	2.50E-01	1.00E+00
KEGG_PATHWAY	Retrograde endocannabinoid signaling	13	3.40E-01	1.00E+00
KEGG_PATHWAY	Toxoplasmosis	13	4.50E-01	1.00E+00
KEGG_PATHWAY	AMPK signaling pathway	13	5.40E-01	1.00E+00
KEGG_PATHWAY	Spliceosome	13	6.40E-01	1.00E+00
KEGG_PATHWAY	Ubiquitin mediated proteolysis	13	7.10E-01	1.00E+00
KEGG_PATHWAY	Measles	13	7.50E-01	1.00E+00
KEGG_PATHWAY	Non-alcoholic fatty liver disease (NAFLD)	13	8.80E-01	1.00E+00
KEGG_PATHWAY	MicroRNAs in cancer	13	1.00E+00	1.00E+00
KEGG_PATHWAY	Central carbon metabolism in cancer	12	4.00E-02	7.80E-01
KEGG_PATHWAY	Pancreatic cancer	12	5.40E-02	7.80E-01
KEGG_PATHWAY	Fc epsilon RI signaling pathway	12	6.50E-02	7.80E-01
KEGG_PATHWAY	B cell receptor signaling pathway	12	8.40E-02	7.80E-01
KEGG_PATHWAY	Melanoma	12	9.80E-02	7.80E-01
KEGG_PATHWAY	Arachidonic acid metabolism	12	9.80E-02	7.80E-01
KEGG_PATHWAY	Chronic myeloid leukemia	12	1.10E-01	7.90E-01
KEGG_PATHWAY	Prolactin signaling pathway	12	1.10E-01	8.00E-01
KEGG_PATHWAY	Aldosterone synthesis and secretion	12	1.60E-01	8.50E-01
KEGG_PATHWAY	Salivary secretion	12	2.00E-01	9.00E-01
KEGG_PATHWAY	Glucagon signaling pathway	12	3.40E-01	1.00E+00
KEGG_PATHWAY	Amoebiasis	12	5.60E-01	1.00E+00
KEGG_PATHWAY	Parkinson's disease	12	9.00E-01	1.00E+00
KEGG_PATHWAY	Long-term potentiation	11	1.10E-01	7.90E-01
KEGG_PATHWAY	p53 signaling pathway	11	1.60E-01	8.50E-01
KEGG_PATHWAY	Ribosome biogenesis in eukaryotes	11	2.60E-01	1.00E+00
KEGG_PATHWAY	Protein digestion and absorption	11	3.20E-01	1.00E+00
KEGG_PATHWAY	Hematopoietic cell lineage	11	4.30E-01	1.00E+00
KEGG_PATHWAY	HIF-1 signaling pathway	11	4.90E-01	1.00E+00
KEGG_PATHWAY	Thyroid hormone signaling pathway	11	7.00E-01	1.00E+00
KEGG_PATHWAY	Chagas disease (American trypanosomiasis)	11	7.10E-01	1.00E+00
KEGG_PATHWAY	Natural killer cell mediated cytotoxicity	11	7.40E-01	1.00E+00

KEGG_PATHWAY	Ovarian steroidogenesis	10	5.60E-02	7.80E-01
KEGG_PATHWAY	Fanconi anemia pathway	10	6.90E-02	7.80E-01
KEGG_PATHWAY	VEGF signaling pathway	10	1.30E-01	8.00E-01
KEGG_PATHWAY	Colorectal cancer	10	2.10E-01	9.00E-01
KEGG_PATHWAY	Adipocytokine signaling pathway	10	2.70E-01	1.00E+00
KEGG_PATHWAY	Inositol phosphate metabolism	10	2.70E-01	1.00E+00
KEGG_PATHWAY	Pertussis	10	3.60E-01	1.00E+00
KEGG_PATHWAY	Insulin secretion	10	4.30E-01	1.00E+00
KEGG_PATHWAY	Small cell lung cancer	10	5.00E-01	1.00E+00
KEGG_PATHWAY	Insulin resistance	10	7.90E-01	1.00E+00
KEGG_PATHWAY	Oocyte meiosis	10	8.10E-01	1.00E+00
KEGG_PATHWAY	Lysosome	10	8.90E-01	1.00E+00
KEGG_PATHWAY	Wnt signaling pathway	10	9.40E-01	1.00E+00
KEGG_PATHWAY	Ether lipid metabolism	9	9.00E-02	7.80E-01
KEGG_PATHWAY	Non-small cell lung cancer	9	1.90E-01	8.90E-01
KEGG_PATHWAY	Steroid hormone biosynthesis	9	2.00E-01	9.00E-01
KEGG_PATHWAY	Apoptosis	9	2.70E-01	1.00E+00
KEGG_PATHWAY	Renin secretion	9	3.30E-01	1.00E+00
KEGG_PATHWAY	Chemical carcinogenesis	9	4.00E-01	1.00E+00
KEGG_PATHWAY	Complement and coagulation cascades	9	4.60E-01	1.00E+00
KEGG_PATHWAY	TGF-beta signaling pathway	9	5.90E-01	1.00E+00
KEGG_PATHWAY	Peroxisome	9	6.10E-01	1.00E+00
KEGG_PATHWAY	Fc gamma R-mediated phagocytosis	9	6.20E-01	1.00E+00
KEGG_PATHWAY	NF-kappa B signaling pathway	9	7.00E-01	1.00E+00
KEGG_PATHWAY	Morphine addiction	9	7.20E-01	1.00E+00
KEGG_PATHWAY	Melanogenesis	9	7.80E-01	1.00E+00
KEGG_PATHWAY	Carbon metabolism	9	8.60E-01	1.00E+00
KEGG_PATHWAY	Cell cycle	9	9.40E-01	1.00E+00
KEGG_PATHWAY	Phagosome	9	9.90E-01	1.00E+00
KEGG_PATHWAY	NOD-like receptor signaling pathway	8	2.40E-01	9.70E-01
KEGG_PATHWAY	Endometrial cancer	8	2.40E-01	9.70E-01
KEGG_PATHWAY	Acute myeloid leukemia	8	3.20E-01	1.00E+00
KEGG_PATHWAY	Metabolism of xenobiotics by cytochrome P450	8	3.70E-01	1.00E+00
KEGG_PATHWAY	Renal cell carcinoma	8	4.90E-01	1.00E+00
KEGG_PATHWAY	Thyroid hormone synthesis	8	5.40E-01	1.00E+00
KEGG_PATHWAY	Gastric acid secretion	8	5.80E-01	1.00E+00
KEGG_PATHWAY	RNA degradation	8	6.40E-01	1.00E+00
KEGG_PATHWAY	Salmonella infection	8	7.30E-01	1.00E+00
KEGG_PATHWAY	ECM-receptor interaction	8	7.80E-01	1.00E+00
KEGG_PATHWAY	Rheumatoid arthritis	8	8.50E-01	1.00E+00
KEGG_PATHWAY	Mucin type O-Glycan biosynthesis	7	9.20E-02	7.80E-01
KEGG_PATHWAY	Bladder cancer	7	2.00E-01	9.00E-01
KEGG_PATHWAY	Proteasome	7	3.10E-01	1.00E+00
KEGG_PATHWAY	Glutathione metabolism	7	4.50E-01	1.00E+00
KEGG_PATHWAY	Lysine degradation	7	4.50E-01	1.00E+00

KEGG_PATHWAY	Amyotrophic lateral sclerosis (ALS)	7	4.50E-01	1.00E+00
KEGG_PATHWAY	Glycerolipid metabolism	7	6.10E-01	1.00E+00
KEGG_PATHWAY	Synaptic vesicle cycle	7	6.10E-01	1.00E+00
KEGG_PATHWAY	Biosynthesis of amino acids	7	7.20E-01	1.00E+00
KEGG_PATHWAY	Hypertrophic cardiomyopathy (HCM)	7	8.20E-01	1.00E+00
KEGG_PATHWAY	Dilated cardiomyopathy	7	8.70E-01	1.00E+00
KEGG_PATHWAY	Tight junction	7	8.80E-01	1.00E+00
KEGG_PATHWAY	Toll-like receptor signaling pathway	7	9.60E-01	1.00E+00
KEGG_PATHWAY	DNA replication	6	2.90E-01	1.00E+00
KEGG_PATHWAY	Nicotine addiction	6	4.10E-01	1.00E+00
KEGG_PATHWAY	Legionellosis	6	6.80E-01	1.00E+00
KEGG_PATHWAY	Arrhythmogenic right ventricular cardiomyopathy (ARVC)	6	7.90E-01	1.00E+00
KEGG_PATHWAY	Amphetamine addiction	6	8.10E-01	1.00E+00
KEGG_PATHWAY	Adherens junction	6	8.20E-01	1.00E+00
KEGG_PATHWAY	Viral myocarditis	6	8.30E-01	1.00E+00
KEGG_PATHWAY	PPAR signaling pathway	6	8.40E-01	1.00E+00
KEGG_PATHWAY	Inflammatory bowel disease (IBD)	6	8.40E-01	1.00E+00
KEGG_PATHWAY	Leishmaniasis	6	8.50E-01	1.00E+00
KEGG_PATHWAY	RIG-I-like receptor signaling pathway	6	8.90E-01	1.00E+00
KEGG_PATHWAY	Bacterial invasion of epithelial cells	6	8.90E-01	1.00E+00
KEGG_PATHWAY	GABAergic synapse	6	9.40E-01	1.00E+00
KEGG_PATHWAY	Systemic lupus erythematosus	6	1.00E+00	1.00E+00
**GOTERM biological processes using default *Bos. taurus* background gene list of DAVID**				
GOTERM_BP_DIRECT	G-protein coupled receptor signaling pathway	84	4.80E-01	1.00E+00
GOTERM_BP_DIRECT	transcription, DNA-templated	77	1.20E-01	1.00E+00
GOTERM_BP_DIRECT	regulation of transcription, DNA-templated	60	8.50E-01	1.00E+00
GOTERM_BP_DIRECT	positive regulation of transcription from RNA polymerase II promoter	51	9.80E-01	1.00E+00
GOTERM_BP_DIRECT	negative regulation of transcription from RNA polymerase II promoter	42	8.20E-01	1.00E+00
GOTERM_BP_DIRECT	intracellular signal transduction	35	2.70E-01	1.00E+00
GOTERM_BP_DIRECT	signal transduction	35	9.60E-01	1.00E+00
GOTERM_BP_DIRECT	cell differentiation	31	1.70E-01	1.00E+00
GOTERM_BP_DIRECT	inflammatory response	30	1.50E-01	1.00E+00
GOTERM_BP_DIRECT	innate immune response	30	2.30E-01	1.00E+00
GOTERM_BP_DIRECT	sensory perception of smell	27	2.10E-01	1.00E+00
GOTERM_BP_DIRECT	protein homooligomerization	26	3.80E-03	1.00E+00
GOTERM_BP_DIRECT	translation	25	1.30E-01	1.00E+00
GOTERM_BP_DIRECT	negative regulation of cell proliferation	25	5.30E-01	1.00E+00
GOTERM_BP_DIRECT	negative regulation of transcription, DNA-templated	25	8.30E-01	1.00E+00
GOTERM_BP_DIRECT	DNA repair	24	2.30E-03	1.00E+00

GOTERM_BP_DIRECT	positive regulation of ERK1 and ERK2 cascade	24	1.50E-02	1.00E+00
GOTERM_BP_DIRECT	cell proliferation	24	4.60E-02	1.00E+00
GOTERM_BP_DIRECT	cell adhesion	24	2.00E-01	1.00E+00
GOTERM_BP_DIRECT	intracellular protein transport	24	2.10E-01	1.00E+00
GOTERM_BP_DIRECT	negative regulation of apoptotic process	24	8.30E-01	1.00E+00
GOTERM_BP_DIRECT	small GTPase mediated signal transduction	23	5.30E-01	1.00E+00
GOTERM_BP_DIRECT	spermatogenesis	22	5.10E-01	1.00E+00
GOTERM_BP_DIRECT	apoptotic process	22	6.60E-01	1.00E+00
GOTERM_BP_DIRECT	immune response	22	7.50E-01	1.00E+00
GOTERM_BP_DIRECT	positive regulation of transcription, DNA-templated	22	9.40E-01	1.00E+00
GOTERM_BP_DIRECT	positive regulation of cell proliferation	22	9.50E-01	1.00E+00
GOTERM_BP_DIRECT	cell surface receptor signaling pathway	21	2.40E-01	1.00E+00
GOTERM_BP_DIRECT	regulation of transcription from RNA polymerase II promoter	21	9.40E-01	1.00E+00
GOTERM_BP_DIRECT	protein autophosphorylation	19	9.00E-02	1.00E+00
**Pathways classified for gene list common between this study, and previous transcriptomics and proteomics studies on puberty of Brahman heifers by our group**				
KEGG_PATHWAY	Metabolic pathways	48	3.40E-03	1.90E-01
KEGG_PATHWAY	Pathways in cancer	16	9.30E-02	6.50E-01
KEGG_PATHWAY	Biosynthesis of antibiotics	15	9.00E-04	1.90E-01
KEGG_PATHWAY	Proteoglycans in cancer	14	2.30E-03	1.90E-01
KEGG_PATHWAY	Ras signaling pathway	12	4.20E-02	4.30E-01
KEGG_PATHWAY	PI3K-Akt signaling pathway	12	2.90E-01	9.30E-01
KEGG_PATHWAY	Calcium signaling pathway	11	2.50E-02	3.20E-01
KEGG_PATHWAY	cAMP signaling pathway	11	3.30E-02	4.00E-01
KEGG_PATHWAY	Oxytocin signaling pathway	10	1.30E-02	2.80E-01
KEGG_PATHWAY	Chemokine signaling pathway	10	5.10E-02	4.40E-01
KEGG_PATHWAY	Neuroactive ligand-receptor interaction	10	3.50E-01	9.90E-01
KEGG_PATHWAY	Circadian entrainment	9	3.60E-03	1.90E-01
KEGG_PATHWAY	Vascular smooth muscle contraction	9	1.20E-02	2.80E-01
KEGG_PATHWAY	FoxO signaling pathway	9	2.10E-02	3.20E-01
KEGG_PATHWAY	Protein processing in endoplasmic reticulum	9	7.30E-02	5.50E-01
KEGG_PATHWAY	Focal adhesion	9	1.70E-01	8.20E-01
KEGG_PATHWAY	Regulation of actin cytoskeleton	9	1.90E-01	8.30E-01
KEGG_PATHWAY	Rap1 signaling pathway	9	1.90E-01	8.30E-01
KEGG_PATHWAY	Estrogen signaling pathway	8	1.40E-02	2.80E-01
KEGG_PATHWAY	Retrograde endocannabinoid signaling	8	1.80E-02	3.20E-01
KEGG_PATHWAY	Carbon metabolism	8	2.30E-02	3.20E-01
KEGG_PATHWAY	Platelet activation	8	4.70E-02	4.30E-01
KEGG_PATHWAY	Dopaminergic synapse	8	4.90E-02	4.30E-01

KEGG_PATHWAY	Adrenergic signaling in cardiomyocytes	8	6.30E-02	5.20E-01
KEGG_PATHWAY	Purine metabolism	8	1.70E-01	8.20E-01
KEGG_PATHWAY	MAPK signaling pathway	8	4.90E-01	1.00E+00
KEGG_PATHWAY	Long-term depression	7	4.10E-03	1.90E-01
KEGG_PATHWAY	Glioma	7	6.60E-03	2.20E-01
KEGG_PATHWAY	Long-term potentiation	7	6.60E-03	2.20E-01
KEGG_PATHWAY	GnRH signaling pathway	7	2.40E-02	3.20E-01
KEGG_PATHWAY	ErbB signaling pathway	7	2.40E-02	3.20E-01
KEGG_PATHWAY	Glutamatergic synapse	7	7.30E-02	5.50E-01
KEGG_PATHWAY	Axon guidance	7	1.10E-01	6.70E-01
KEGG_PATHWAY	Hepatitis C	7	1.30E-01	7.20E-01
KEGG_PATHWAY	cGMP-PKG signaling pathway	7	2.30E-01	9.00E-01
KEGG_PATHWAY	Influenza A	7	2.90E-01	9.30E-01
KEGG_PATHWAY	Alzheimer's disease	7	3.20E-01	9.70E-01
KEGG_PATHWAY	Tuberculosis	7	3.30E-01	9.70E-01
KEGG_PATHWAY	Biosynthesis of amino acids	6	3.70E-02	4.20E-01
KEGG_PATHWAY	Prostate cancer	6	7.20E-02	5.50E-01
KEGG_PATHWAY	Glycerophospholipid metabolism	6	1.10E-01	6.70E-01
KEGG_PATHWAY	Inflammatory mediator regulation of TRP channels	6	1.30E-01	7.20E-01
KEGG_PATHWAY	Serotonergic synapse	6	1.80E-01	8.30E-01
KEGG_PATHWAY	Neurotrophin signaling pathway	6	2.20E-01	8.90E-01
KEGG_PATHWAY	Spliceosome	6	2.40E-01	9.00E-01
KEGG_PATHWAY	Ribosome	6	2.70E-01	9.20E-01
KEGG_PATHWAY	Parkinson's disease	6	3.60E-01	9.90E-01
KEGG_PATHWAY	Huntington's disease	6	5.90E-01	1.00E+00
KEGG_PATHWAY	Cytokine-cytokine receptor interaction	6	6.70E-01	1.00E+00
KEGG_PATHWAY	Viral carcinogenesis	6	7.30E-01	1.00E+00
KEGG_PATHWAY	HTLV-I infection	6	8.30E-01	1.00E+00
KEGG_PATHWAY	Nicotine addiction	5	2.30E-02	3.20E-01
KEGG_PATHWAY	NOD-like receptor signaling pathway	5	4.30E-02	4.30E-01
KEGG_PATHWAY	Amphetamine addiction	5	9.60E-02	6.50E-01
KEGG_PATHWAY	PPAR signaling pathway	5	1.10E-01	6.70E-01
KEGG_PATHWAY	Melanoma	5	1.20E-01	6.90E-01
KEGG_PATHWAY	Chronic myeloid leukemia	5	1.20E-01	6.90E-01
KEGG_PATHWAY	Aldosterone synthesis and secretion	5	1.50E-01	7.50E-01
KEGG_PATHWAY	Progesterone-mediated oocyte maturation	5	1.90E-01	8.30E-01
KEGG_PATHWAY	Morphine addiction	5	2.20E-01	8.90E-01
KEGG_PATHWAY	HIF-1 signaling pathway	5	2.40E-01	9.00E-01
KEGG_PATHWAY	Phosphatidylinositol signaling system	5	2.50E-01	9.00E-01
KEGG_PATHWAY	Pyrimidine metabolism	5	2.50E-01	9.00E-01
KEGG_PATHWAY	T cell receptor signaling pathway	5	2.90E-01	9.30E-01
KEGG_PATHWAY	Cholinergic synapse	5	3.20E-01	9.70E-01
KEGG_PATHWAY	Epstein-Barr virus infection	5	3.70E-01	9.90E-01
KEGG_PATHWAY	Insulin signaling pathway	5	4.70E-01	1.00E+00

KEGG_PATHWAY	Wnt signaling pathway	5	4.80E-01	1.00E+00
KEGG_PATHWAY	Measles	5	5.00E-01	1.00E+00
KEGG_PATHWAY	Cell adhesion molecules (CAMs)	5	5.70E-01	1.00E+00
KEGG_PATHWAY	Phagosome	5	5.90E-01	1.00E+00
KEGG_PATHWAY	Transcriptional misregulation in cancer	5	6.50E-01	1.00E+00
KEGG_PATHWAY	Endocytosis	5	8.80E-01	1.00E+00
KEGG_PATHWAY	2-Oxocarboxylic acid metabolism	4	1.10E-02	2.80E-01
KEGG_PATHWAY	Citrate cycle (TCA cycle)	4	4.20E-02	4.30E-01
KEGG_PATHWAY	Thyroid cancer	4	4.50E-02	4.30E-01
KEGG_PATHWAY	Bladder cancer	4	8.50E-02	6.10E-01
KEGG_PATHWAY	Proteasome	4	1.20E-01	6.90E-01
KEGG_PATHWAY	Ether lipid metabolism	4	1.20E-01	6.90E-01
KEGG_PATHWAY	Ovarian steroidogenesis	4	1.40E-01	7.30E-01
KEGG_PATHWAY	Non-small cell lung cancer	4	1.80E-01	8.20E-01
KEGG_PATHWAY	VEGF signaling pathway	4	2.00E-01	8.30E-01
KEGG_PATHWAY	Glycerolipid metabolism	4	2.20E-01	8.90E-01
KEGG_PATHWAY	Pancreatic cancer	4	2.40E-01	9.00E-01
KEGG_PATHWAY	Fc epsilon RI signaling pathway	4	2.50E-01	9.00E-01
KEGG_PATHWAY	Adherens junction	4	2.60E-01	9.10E-01
KEGG_PATHWAY	Inositol phosphate metabolism	4	2.80E-01	9.30E-01
KEGG_PATHWAY	Adipocytokine signaling pathway	4	2.80E-01	9.30E-01
KEGG_PATHWAY	Antigen processing and presentation	4	3.10E-01	9.60E-01
KEGG_PATHWAY	Pertussis	4	3.20E-01	9.70E-01
KEGG_PATHWAY	Hypertrophic cardiomyopathy (HCM)	4	3.40E-01	9.90E-01
KEGG_PATHWAY	Insulin secretion	4	3.60E-01	9.90E-01
KEGG_PATHWAY	TGF-beta signaling pathway	4	3.70E-01	9.90E-01
KEGG_PATHWAY	Salmonella infection	4	3.70E-01	9.90E-01
KEGG_PATHWAY	Fc gamma R-mediated phagocytosis	4	3.80E-01	1.00E+00
KEGG_PATHWAY	GABAergic synapse	4	3.90E-01	1.00E+00
